# MYC up-regulation confers vulnerability to dual inhibition of CDK12 and CDK13 in high-risk Group 3 medulloblastoma

**DOI:** 10.1186/s13046-023-02790-2

**Published:** 2023-08-21

**Authors:** Consuelo Pitolli, Alberto Marini, Marika Guerra, Marco Pieraccioli, Veronica Marabitti, Fernando Palluzzi, Luciano Giacò, Gianpiero Tamburrini, Francesco Cecconi, Francesca Nazio, Claudio Sette, Vittoria Pagliarini

**Affiliations:** 1 Department of Neuroscience, Section of Human Anatomy, Catholic University of the Sacred Heart, 00168 Rome, Italy; 2 GSTEP-Organoids Research Core Facility, IRCCS Fondazione Policlinico Universitario Agostino Gemelli, 00168 Rome, Italy; 3 Department of Pediatric Hemato-Oncology and Cell and Gene Therapy, Bambino Gesù Children’s Hospital, IRCCS, Rome, Italy; 4 Department of Biology, University of Rome Tor Vergata, Rome, Italy; 5 Bioinformatics Research Core Facility, Gemelli Science and Technology Park (GSTeP), IRCCS Fondazione Policlinico Universitario Agostino Gemelli, 00168 Rome, Italy; 6 Present Address: Integrated Omics Department, Novo Nordisk, 2860 Søborg, Denmark; 7 Pediatric Neurosurgery, IRCCS Fondazione Policlinico Universitario Agostino Gemelli, 00168 Rome, Italy; 8 Department of Basic Biotechnological Sciences, Intensive Care and Perioperative Clinics Research, Catholic University of the Sacred Heart, 00168 Rome, Italy; 9 Unit of Cell Stress and Survival, Danish Cancer Society Research Center, Copenhagen, Denmark

**Keywords:** THZ531, RNA polymerase processivity, RNA processing regulation, Brain tumors, Chemotherapy resistance

## Abstract

**Background:**

Medulloblastoma (MB) is the most common cerebellar malignancy during childhood. Among MB, MYC-amplified Group 3 tumors display the worst prognosis. MYC is an oncogenic transcription factor currently thought to be undruggable. Nevertheless, targeting MYC-dependent processes (i.e. transcription and RNA processing regulation) represents a promising approach.

**Methods:**

We have tested the sensitivity of MYC-driven Group 3 MB cells to a pool of transcription and splicing inhibitors that display a wide spectrum of targets. Among them, we focus on THZ531, an inhibitor of the transcriptional cyclin-dependent kinases (CDK) 12 and 13. High-throughput RNA-sequencing analyses followed by bioinformatics and functional analyses were carried out to elucidate the molecular mechanism(s) underlying the susceptibility of Group 3 MB to CDK12/13 chemical inhibition. Data from International Cancer Genome Consortium (ICGC) and other public databases were mined to evaluate the functional relevance of the cellular pathway/s affected by the treatment with THZ531 in Group 3 MB patients.

**Results:**

We found that pharmacological inhibition of CDK12/13 is highly selective for MYC-high Group 3 MB cells with respect to MYC-low MB cells. We identified a subset of genes enriched in functional terms related to the DNA damage response (DDR) that are up-regulated in Group 3 MB and repressed by CDK12/13 inhibition. Accordingly, MYC- and CDK12/13-dependent higher expression of DDR genes in Group 3 MB cells limits the toxic effects of endogenous DNA lesions in these cells. More importantly, chemical inhibition of CDK12/13 impaired the DDR and induced irreparable DNA damage exclusively in MYC-high Group 3 MB cells. The augmented sensitivity of MYC-high MB cells to CDK12/13 inhibition relies on the higher elongation rate of the RNA polymerase II in DDR genes. Lastly, combined treatments with THZ531 and DNA damage-inducing agents synergically suppressed viability of MYC-high Group 3 MB cells.

**Conclusions:**

Our study demonstrates that CDK12/13 activity represents an exploitable vulnerability in MYC-high Group 3 MB and may pave the ground for new therapeutic approaches for this high-risk brain tumor.

**Supplementary Information:**

The online version contains supplementary material available at 10.1186/s13046-023-02790-2.

## Background

Medulloblastoma (MB) is a tumor of the cerebellum and represents the most common brain cancer in pediatric patients. Current treatments for MB are based on a combination of chemotherapy, surgical resection and cranio-spinal irradiation, which are not specific for this cancer and only partially improve the survival of patients. Furthermore, these aggressive treatments can lead to neurocognitive deficit in children and adolescents [1]. Thus, there is an urgent need to identify novel therapies that specifically target tumor cells, while limiting damage to healthy brain tissue.

MB comprises four subgroups named Wingless (WNT), Sonic Hedgehog (SHH), Group 3 and Group 4 [1]. These subgroups are characterized by different histological, genetic and molecular features, as well as clinical outcome [1]. The Group 3 accounts for 25% of all MBs and is associated with the highest rate of metastasis at diagnosis and the worst survival outcome at 5 years [1]. A frequent feature of Group 3 MB (~ 17% of patients) is the amplification of the *MYC* oncogene, which promotes malignancy and associates with poor clinical outcome [1]. Up-regulation of MYC is considered a driver for the onset and progression of many human cancers, including Group 3 MB. Unfortunately, no specific drug is currently available to directly target MYC [2]. However, MYC-dependent transcriptional outputs might represent a promising target to indirectly impact downstream of MYC activity and identification of critical targetable nodes in the MYC pathway might pave the ground for the development of more efficacious and less toxic anticancer therapies.

Transcription-associated (TA) cyclin-dependent kinases (CDKs) are emerging as promising actionable targets in cancer [3]. TA-CDKs phosphorylate the carboxyl-terminal domain (CTD) of the RNA polymerase II (RNAPII) largest subunit and modulate its function by orchestrating the sequential recruitment of co-factors that are required at different steps of the transcription cycle [4]. CDK7, CDK8 and CDK9 are involved at early stages [5], and determine the transition of the RNAPII from promoter proximal pausing to elongation. On the other hand, CDK12 and CDK13 promote transcription elongation within the gene body, thus regulating the processivity of RNAPII [6]. Moreover, CDK12/13 were proposed to couple transcription elongation with pre-mRNA processing events, such as splicing and polyadenylation [3, 5]. Accordingly, CDK12/13 inhibition elicits genome-wide transcriptome changes by increasing the retention of introns and causing early termination at proximal polyadenylation sites [6, 7]. CDK12/13 activity is particularly important in cells with high transcriptional rate, such as cancer cells harboring MYC amplification or up-regulation [3]. Recent studies have described gene-selective and non-overlapping roles for CDK12 and CDK13. In particular, genetic depletion or pharmacologic inhibition of CDK12 predominantly affected the expression of DNA damage response (DDR) genes [6, 8–11], thus explaining the BRCA-like phenotype observed in human cancers harbouring mutations in the *CDK12* gene [3, 12] and the synergic effect of CDK12 inhibitors administered in combination with PARP inhibitors [13–15]. However, a substantial functional redundancy between CDK12/13 has also been demonstrated [6], with much stronger effects on gene expression being observed upon concomitant inhibition of both kinases [6, 7, 16]. These findings suggested that CDK12 and CDK13 act in a cooperative manner to regulate RNAPII dynamics across the genome and to prevent early termination at cryptic polyadenylation sites. Nevertheless, whether CDK12/13 play an important role also in *MYC*-amplified Group 3 MB is currently unknown.

Several inhibitors of TA-CDKs were shown to downregulate MYC expression, to suppress growth of MB cells and to sensitize them to chemotherapeutic agents [17–20]. Moreover, *MYC*-amplified cancer cells were reported to be particularly sensitive also to splicing inhibitors [21]. Herein, by screening multiple TA-CDK and splicing inhibitors, we found that MYC-driven Group 3 MB cell lines exhibited high sensitivity to the CDK12/13 inhibitor THZ531. Pharmacologic inhibition of CDK12/13 strongly reduced the expression of DDR genes, leading to irreparable DNA damage and massive apoptotic cell death in MYC-high Group 3 MB cells. Furthermore, we provide evidence that the augmented sensitivity of MYC-high Group 3 MB cells to CDK12/13 inhibition relies on the higher elongation rate of RNAPII in DDR genes, which is associated with the higher expression of DDR proteins required for limiting endogenous DNA lesions. We also provide a mechanistic insight on this high dependency by showing that MYC-high MB cells strongly depend on an efficient DDR pathway for their survival and that CDK12/13 activity is essential to guarantee it. Lastly, treatment with THZ531 synergically enhanced the cytotoxic effects of DNA damage-inducing agents in Group 3 MB cells. Thus, our study points to CDK12/13 activity as an exploitable vulnerability for this high-risk brain tumor, for which no targeted therapies are currently available.

## Methods

### Human cell lines

DAOY, D283-Med (hereafter referred to as D283) and D341-Med (hereafter referred to as D341) cells were cultured according to the recommended conditions (ATCC). DAOY and D283 cells were cultured in Minimal Essential Medium (MEM, Gibco) supplemented with sodium pyruvate 1 mM (Gibco), MEM Non-Essential Amino Acids Solution 1X (Gibco), 10% fetal bovine serum (FBS, Gibco), 100 U/ml penicillin and 100 μg/ml streptomycin (Euroclone). For D341 cell line additional 10% FBS was used. Primary human MB cells MED-411-FH (hereafter referred to as MED-411) are PDX cell lines which were generated by the James M. Olson laboratory [22] and were cultured in NeuroCult NS-A basal medium with NeuroCult NS-A Proliferation Supplements (Stem Cell Technologies), Epidermal Growth Factor 20 ng/ml (EGF, Peprotech), Fibroblast Growth Factor 20 ng/ml (FGF, Peprotech), 100 U/ml penicillin and 100 μg/ml streptomycin (Euroclone). ONS-76 (gently provided by Dr Giampiero di Leva, Keele University, UK) and HD-MBO3 (obtained from Deutsche Sammlung von Mikroorganismen und Zellkulturen DSMZ, Germany) were maintained in RPMI (Gibco) with 10% FBS (Gibco), 100 U/ml penicillin and 100 μg/ml streptomycin (Euroclone). HEK293T cells were maintained in Dulbecco-Minimal Essential Medium (DMEM, Sigma-Aldrich) supplemented with MEM Non-Essential Amino Acids Solution 1X (Gibco), 10% FBS (Gibco), 100 U/ml penicillin and 100 μg/ml streptomycin (Euroclone). All cell lines were cultured at 37 °C in humidified atmosphere with 5% CO_2_ and tested for mycoplasma contamination by PCR every 3 months.

### Cell viability assay

Cell viability was assessed by CellTiter 96® AQueous One Solution Cell Proliferation Assay ([3-(4,5-dimethylthiazol-2-yl)-5-(3-carboxymethoxyphenyl)-2-(4-sulfophenyl)-2H-tetrazolium, inner salt; MTS, Cat. No. G3582 Promega]. Cells were seeded in 96-well plates and treated for 72 h with EPZ015666 (Cat. No. HY-12727, MedChemExpress), *Pladienolide B* (Cat. No. sc-391691, Santa Cruz Biotechnology), Indisulam (Cat. No. HY-13650, MedChemExpress), Dinaciclib (Cat. No. HY-10492, MedChemExpress), THZ1 (Cat. No. HY-80013, MedChemExpress), THZ531 (Cat. No. HY-103618, MedChemExpress), SR4835 (Cat. No. HY-130250, MedChemExpress), Cisplatin (Cat. No: 1166, Selleckchem), Olaparib (Cat. No. HY-10162, MedChemExpress), ATM kinase inhibitor (Cat. No. 118502 Sigma-Aldrich) and ATR kinase inhibitor/Cerelasertib (Cat. No. HY-19323 MedChemExpress) at the indicated doses. The assay was performed by adding 20 μl of the CellTiter 96® AQueous One Solution Reagent directly to culture wells, incubating for 2 h and then recording absorbance at 490 nm with a 96-well plate reader (iMark™ Microplate Absorbance Reader, BioRad). The quantity of formazan product as measured by the amount of 490 nm absorbance was directly proportional to the number of living cells in culture.

For quantification of cell confluence, cells were seeded in a 96 well plate and imaged at 10X magnification in an IncuCyte SX5 Live-content imaging system (Essen Bioscience) at 37 °C with 5% CO_2_. The images were acquired every 12 h for the indicated hours (4 images/well) and analysed using IncuCyte Cell-by-Cell analysis software.

### Western blot analysis and antibodies

Total proteins were extracted from cells by using a lysis buffer (150 mM NaCl, 15 mM MgCl_2_, 15 mM EGTA, 50 mM HEPES pH 7.4, 20 mM β-glycerophosphate, 10% glycerol, 1% Triton X-100) supplemented with 1% protease inhibitor cocktail (Sigma Aldrich), 0.5 mM Na_3_VO_4_, 1 mM DTT. Extracted proteins were resolved on 10% SDS-PAGE gels (20 μg/lane) and transferred onto nitrocellulose membrane (Amersham). Blots were incubated with the indicated primary antibody in 5% non-fat dry milk in PBS plus 0.1% Tween-20 overnight at 4 °C. Primary antibodies used were: anti-BRCA1 (Cat. No. BK9010S, Cell Signaling); anti-RAD51 (Cat. No. PC130, Millipore); anti-pH2AX (Ser139) (Cat. No. 9718, Cell Signaling); anti-PARP (Cat. No. 9402S, Cell Signaling); anti-c-MYC (Cat. No. K9402S, Cell Signaling); anti-HSP90 (Cat. No. sc-13119, Santa Cruz Biotechnology). Detection was achieved by using anti-mouse HRP-conjugated (Cat. No. NA931, Amersham) and anti-rabbit HRP-conjugated (Cat. No. NA934, Amersham) and visualized by Clarity Western ECL Substrate (Cat. No. 1705061, Bio-Rad).

### RNA extraction and real-time qPCR analyses

Total RNA was extracted by using Trizol reagent (Invitrogen) according to standard procedures [23]. After digestion with RNase-free DNase (Roche), total RNA was retro-transcribed with random primers using M-MLV reverse transcriptase (Promega) according to standard procedures [23]. qPCR was carried out using LightCycler 480 SYBR Green I Master and the LightCycler 480 System (Roche), according to manufacturer's instructions [23]. Primers used in RT-PCR and qPCR experiments are listed in Additional file 1: Supplementary Table 1.

### Cell transfections

Cells were transfected with 50 nM of specific siRNAs by Lipofectamine RNAiMax Transfection Reagent (Cat. No. 13778150, Invitrogen). A pool of 4 siRNAs against c-Myc was used to silence MYC expression (Cat. No. L-003282–0, Dharmacon, Lafayette, CO, USA). Non-targeting scrambled siRNAs were used as negative control. RNA and proteins were extracted 48 h after transfection. The sequences of siRNAs used in knockdown experiments are listed in Additional file 1: Supplementary Table 1.

### Lentiviral vectors construction and transduction

Short hairpin (shRNA) sequence targeting c-MYC or Non-Target control shRNA were cloned in pLVTHM vector by using MluI and ClaI restriction enzymes. For lentivirus particles production, constructs (5 μg) were transfected in the presence of psPAX2 (2.5 μg) and pMD2.G (2 μg) packaging vectors into HEK293T cells by using JetPrime reagent (Cat. No. 101000046 Polyplus). After 48 h, the supernatant containing lentiviral particles was collected and centrifuged at 3.000 rpm for 5 min. The lentivirus was stored in aliquots at − 80 °C. Lentiviral particles were used to infect MED-411 cells for 24 h in the presence of Polybrene (10 μg/ml).

### FACS analysis

Cells were fixed in 50% methanol/acetone 4:1 mix for 30 min at + 4 °C, then treated with 10 μg/ml of RNase A for 20 min at 37 °C and stained with 20 μg/ml of propidium iodide for 20 min. Twelve thousand events were acquired using CytoFlex flow cytometer (Beckman) and analyzed using FlowJo v.10 software (Becton Dickinson).

### DNA damage evaluation by comet assay

DNA damage was evaluated by comet assay (single cell gel electrophoresis) under denaturing conditions [24]. Briefly, dust-free frosted-end microscope slides were kept in methanol overnight to remove fatty residues. Slides were then dipped into molten Normal Melting Point (NMP) agarose at 1% and left to dry. Cell pellets were resuspended in PBS and kept on ice to inhibit DNA repair. Cell suspensions were mixed with Low Melting Point (LMP) agarose at 0.5% kept at 37° C and an aliquot was pipetted onto agarose-covered surface of the slide. Agarose embedded cells were lysed by submerging slides in lysis solution (2.5 M NaCl, 100 mM EDTA, 10 mM Tris base pH 10.0). Slides were then equilibrated for 20 min in running buffer (300 mM NaOH, 1 mM EDTA pH 13.0). Electrophoresis was performed for 20 min at 0.6 V/cm. Slides were subsequently neutralized with 0.4 M Tris–HCl, washed in distilled water and finally dehydrated in ice cold methanol. Nuclei were stained with GelRed (1:1000) and visualized with a fluorescence microscope (Zeiss), using a 40X objective, connected to a CCD camera for image acquisition. At least 200 comets per each experimental point were analyzed using TriTek Comet Score software and data from tail moments processed using Prism software. Apoptotic cells were excluded from the analysis to avoid artificial enhancement of the tail moment.

### RNA-seq analysis

The GSE225375 dataset was analyzed for gene expression. Quality reads repartition (e.g., for potential ribosomal contamination), inner distance size estimation, gene body coverage, strand-specificity of library were performed using FastQC v0.11.2, Picard-Tools v1.119, Samtools v1.0, and RSeQC v2.3.9. Reads were mapped using STAR v2.4.0f1 [25] on the human hg19 genome assembly and read count was performed using feature Count from SubRead v1.5.0. Read counts were normalized using DESeq2 [26] and ERCC spike-in as control gene. Gene expression estimation was performed as described previously [27] using Human FAST DB v2018_1 annotations. Genes were considered as expressed if their FPKM value was greater than FPKM of 98% of the intergenic regions (background) and only genes expressed in at least one of the two compared conditions were evaluated. For differential expressed, we used as thresholds fold-change ≥ 1.5 or 2 and *p*-value ≤ 0.05.

Distribution of gene lengths and FPKM were compared between down-regulated, up-regulated and unregulated genes in D341 treated with 200 nM THZ531 or DMSO for 6 h using the Student’s t test with ‘holm’ method for adjusting *p* values of ‘ggpubr’ package in R.

Gene metaprofile was created by dividing each gene from transcription star site (TSS) to transcription end site (TES) into 50 equally sized bins. Bam files were used to calculate read coverage (read counts per million mapped reads) between down-regulated and up-regulated genes in D341 treated with 200 nM THZ531 or DMSO for 6 h in strand aware mode using ‘Genomation’ package in R. To account for the differential gene expression, reads counts were rescaled as percentage of mapped reads and their distribution within the gene body was drawn using ‘ggplot2’ package in R.

### Gene expression analysis in Group 3 MB patients

EGA RNA-seq expression dataset (EGAD00001004958; Application #: DACO-5889) was firstly inspected through principal components analysis (PCA) to evaluate the separation among Group 3 MB, healthy fetal (HFC) and adult (HAC) cerebellum samples [28]. RNA-seq data were counted per million mapped reads (CPM)-normalized and non-expressed genes were removed from the dataset. Differential expression analysis was then applied using empirical Bayesian methods, generating log_2_ fold-changes, *p*-values and false discovery rates (FDR, corresponding to Benjamini–Hochberg adjusted *p*-values) for each gene [29]. An FDR < 0.05 defined differentially expressed genes (DEGs). Among DEGs, we identified a set of co-regulated (i.e. same direction of regulation) genes across different comparisons. These genes were referred to as common DEGs. Log_2_ RNA-seq counts were then standardized (mean-subtracted and divided by the standard deviation) then clustered using a k-means algorithm, with k = 4 [28]. To reduce the effect of the initial k-means random seed assignment (i.e. different resulting clusters), we generated a perturbation score corresponding to the negative log_10_ of the combined *p*-value of all the comparisons for each gene. The gene-wise combined *p*-value is obtained using Fisher’s sum of log *p*-values (sumlog method; R package version 1.8). Combined FDRs are then generated from nominal combined *p*-values. The perturbation score is the negative log_10_ of the combined FDR. Common DEGs could be then predicted using a combined score optimal threshold generated by maximizing sensitivity and specificity. Only DEGs with a combined score above the optimal threshold were retained and used for clustering. This procedure provided an objective way to retain top-regulated DEGs among comparisons, reducing cluster sizes and leading to clusters that are much less sensitive to the initial k-means seeds choice. An agglomerative hierarchical clustering (average linkage) [28] on columns was done to assess a perfect separation among MB, HFC and HAC groups. Each cluster was further characterized through an over-representation analysis (ORA) over KEGG, Reactome and Gene Ontology (biological process, molecular function and cellular component) using the online tool Enrichr [30].

Statistical analyses were evaluated by unpaired Student's t-test or One-Way or Two-Way ANOVA test, as indicated.

## Results

### MYC confers susceptibility of Group 3 MB cells to pharmacological inhibition of CDK12/13

Amplification of the oncogene *MYC* is a frequent feature of Group 3 MB [31]. Since over-expression of MYC in cancer cells exposes them to higher vulnerability to splicing inhibition [21], we set out to investigate the cytotoxicity of drugs that affect different steps of the splicing process. To this end, we employed three MB cell lines that express either low (DAOY; SHH MB) or high (D283 and D341; Group 3 MB) MYC levels (Additional file 2: Supplementary Fig. 1A). Viability assays were carried out in cells treated with increasing doses of drugs that directly or indirectly target the splicing machinery: EPZ015666, an inhibitor of PRMT5 which catalyzes asymmetric dimethylation of Sm proteins and promotes assembly of the spliceosomal small nuclear ribonucleoproteins (snRNPs; [32]); Pladienolide B, which inhibits the U2snRNP protein SF3B1 and affects the first catalytic step of the splicing reaction [33]; Indisulam, which leads to degradation of the RNA binding protein (RBP) RMB39 that is involved in recognition of the 3' splice site [34]; the CDK inhibitors Dinaciclib [35], THZ1 [36] and THZ531 [13], which all reduce phosphorylation of the RNAPII CTD, thus affecting both transcription and pre-mRNA processing [3, 12, 37]. We found that the MB cell lines were relatively resistant to PRMT5 inhibition (Additional file 2: Supplementary Fig. 1B,C), while all being equally sensitive to Pladienolide B, Indisulam and THZ1 (Additional file 2: Supplementary Fig. 1B,D,E,G), regardless of their MYC status. By contrast, treatment with low nanomolar concentrations of Dinaciclib (10–50 nM) and THZ531 (50–100 nM) mainly affected the viability of MYC-high D283 and D341 cells (Additional file 2: Supplementary Fig. 1B,F,H), with THZ531 having the greatest differential effect. THZ531 is a specific inhibitor of CDK12/13 [13], while Dinaciclib is a pan-CDK inhibitor that was also shown to inhibit CDK12 [38]. Moreover, D283 and D341 cells exhibited increased sensitivity also to SR4835 (Additional file 2: Supplementary Fig. 1B,I), another CDK12/13 inhibitor with a different structure with respect to THZ531 [39]. Thus, we focused on CDK12/13 as potential specific targets of MYC-high MB cells.

**Fig. 1:**
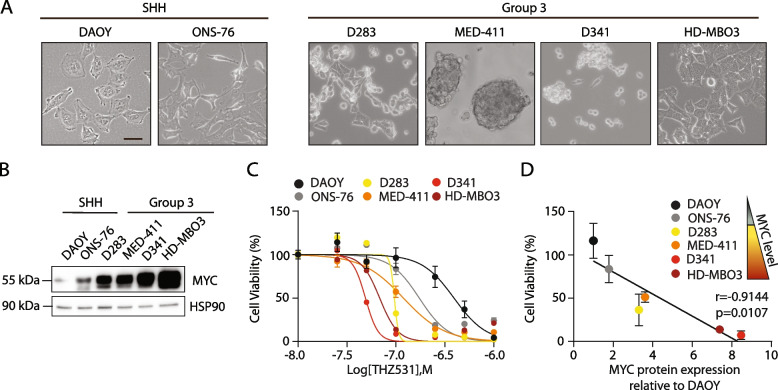
MYC confers susceptibility of Group 3 MB cells to pharmacological inhibition of CDK12/13. **A** Representative images of SHH and Group 3 MB cell lines used in this study. Scale bar: 50 μm. **B** WB analysis of MYC expression level in MB cells shown in A. HSP90 was used as loading control. Blots are representative of 3 independent experiments. **C** MTS assay of MB cells treated for 72 h with increasing doses of THZ531, as indicated. Data represent mean ± SEM of 3 independent experiments. **D** Pearson’s correlation between MYC protein expression levels and MB cell viability (%) following treatment with 100 nM THZ531 for 72 h. Pearson’s correlation coefficient (r) and *p*-value (p) are reported. Data relative to cell viability are reported as mean ± SEM of 3 independent experiments and MYC protein expression is expressed as mean value obtained from densitometric analysis of 3 biological replicates

To extend the observation to additional models, we employed two other MYC-high Group 3 MB cells, HD-MBO3 and the patient-derived primary cell line MED-411, and the SHH cell line ONS-76 (Fig. 1A,B). Viability assays indicated that the effect of THZ531 was highly correlated with MYC expression (*r* = 0.9144; *p* = 0.0107), being lowest in the DAOY cell line and greatest in the Group 3 MB cell lines D341 and HD-MBO3 cells (Fig. 1C,D), which express the highest levels of MYC (Fig. 1B). These data suggest that high MYC levels confer vulnerability to CDK12/13 inhibition in MB cells.

### Pharmacologic inhibition of CDK12/13 reprograms the transcriptome of MYC-high Group 3 MB cells

**Fig. 2:**
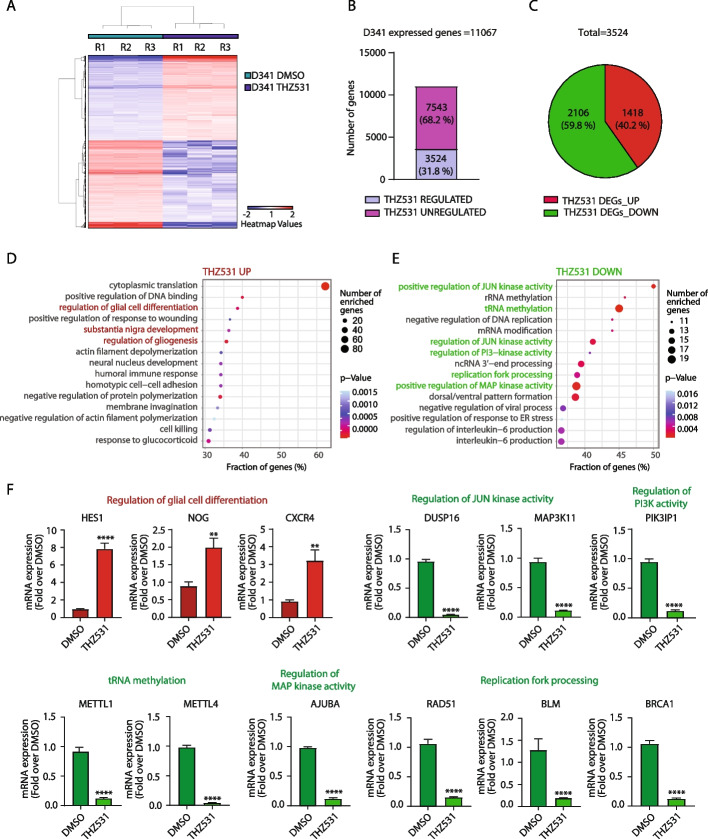
Pharmacologic inhibition of CDK12/13 reprograms the transcriptome of MYC-driven Group 3 MB cells. **A** Heat map of gene expression after treatment with 200 nM THZ531 for 6 h in D341 cell line. FC > 2, p-Adj < 0.05. **B** Bar graph shows the percentage of regulated and unregulated genes in D341 cells upon treatment with THZ531, as above. **C** Pie chart of up-regulated and down-regulated genes (%) in D341 cells upon treatment with THZ531, as above. **D** GO analysis of up-regulated genes performed by using TopGO package in R Studio Software. *P*-value was calculated with Classic Fisher’s Exact test. **E** GO analysis of down-regulated genes performed as above. **F** Bar graphs showing the results of qPCR analyses for the expression of the indicated genes. mRNA expression is normalized on GAPDH expression. Graphs show the mean ± SD of 3 independent experiments. Statistical analysis was performed by unpaired two-tailed Student’s t test (**p < 0.01, ****p < 0.0001)

To investigate the molecular mechanism(s) underlying the susceptibility of Group 3 MB to CDK12/13 chemical inhibition, we performed RNA sequencing (RNA-Seq) analysis in D341 cells treated with THZ531 (Fig. 2A and Additional file 3: Supplementary Table 2). Short-term (6 h) inhibition of CDK12/13 exerted a widespread effect on the transcriptome of D341 cells, with ~ 32% of the expressed genes being modulated (Fig. 2B). While THZ531 preferentially caused downregulation of expression, a substantial fraction of target genes (~ 40%) was also up-regulated by the treatment (Fig. 2C). Gene Ontology (GO) analysis of up-regulated genes highlighted functional categories related to mRNA translation (Fig. 2D and Additional file 6: Supplementary Table 5), possibly indicating the attempt of cells to withstand the transcriptional repression caused by inhibition of CDK12/13 activity by increasing the translation efficiency of existing mRNAs. Up-regulated genes were also enriched for functional categories related to brain differentiation (i.e. substantia nigra development, regulation of glial cell differentiation, regulation of gliogenesis, homotypic cell–cell adhesion; Fig. 2D and Additional file 6: Supplementary Table 5), suggesting that CDK12/13 inhibition induces a switch from proliferation to differentiation in MB cells. On the other hand, down-regulated genes were enriched in terms related to various tumor-associated survival pathways (i.e. positive regulation of JUN kinase activity, tRNA methylation, regulation of JUN kinase activity, regulation of phosphatidylinositol 3 − kinase activity, replication fork processing and positive regulation of MAP kinase activity; Fig. 2E and Additional file 6: Supplementary Table 5). Independent quantitative real time PCR (qPCR) analysis of 22 genes belonging to the principal functional categories highlighted by GO analysis yielded a validation rate of ~ 82% (Fig. 2F and Additional file 2: Supplementary Fig. 2A), thus confirming the reliability of our bioinformatics analysis. Moreover, the majority (80–90%) of the genes tested (*n* = 12) was modulated in the same direction by THZ531 treatment also in three other Group 3 MB cell lines (D283, MED-411 and HD-MBO3, Additional file 2: Supplementary Fig. 2B-D), indicating the reproducibility of the results in multiple Group 3 MB cellular contexts. These findings suggest that pharmacologic inhibition of CDK12/13 reprograms the transcriptome of MYC-high MB cells by increasing the expression of differentiation-related genes, while repressing genes associated to cancer-related survival pathways.

### CDK12/13 inhibition impairs the DNA damage response in MYC-driven Group 3 MB cells

**Fig. 3:**
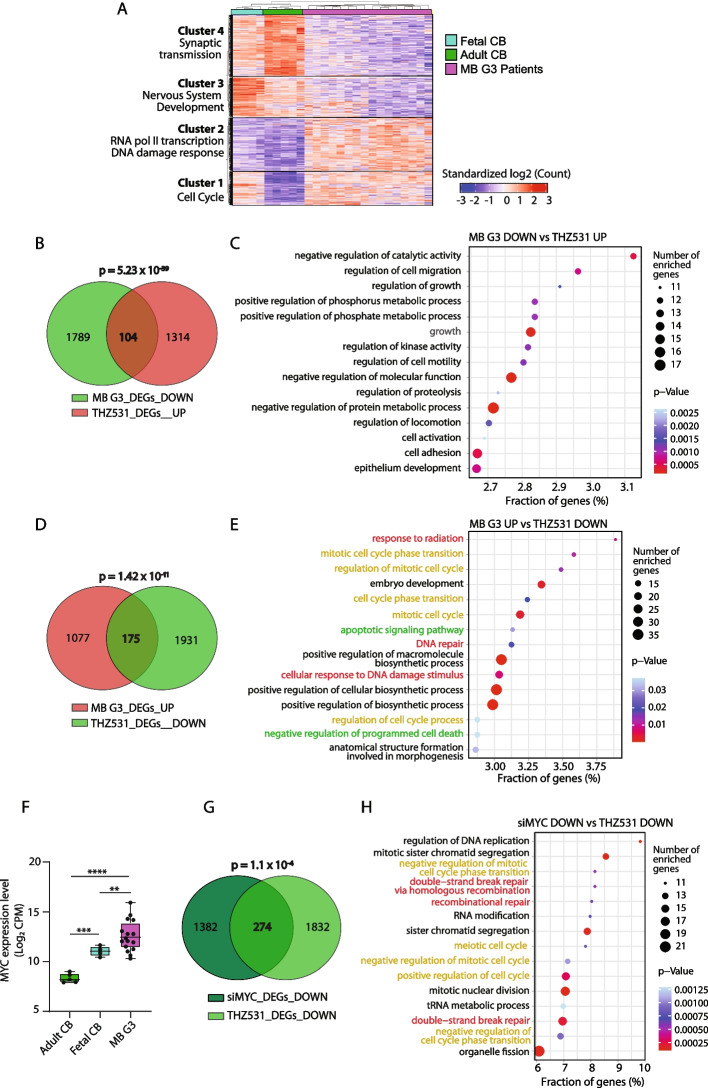
CDK12/13 inhibition impairs the DNA damage response in MYC-driven Group 3 MB cells. **A** Heat map of differentially expressed genes in Group 3 MB patients compared to fetal and adult cerebella. **B** Venn diagram showing a significant overlap between genes down-regulated in Group 3 MB patients compared to healthy controls and genes up-regulated in THZ531-treated D341 cells, as above. Statistical analysis was performed by hypergeometric test using the phyper function of R Stats Package in R Studio Software. **C** GO terms enriched for genes of the overlap shown in D. GO analysis was performed as above. **D** Venn diagram showing a significant overlap between genes up-regulated in Group 3 MB patients compared to healthy controls and genes down-regulated in D341 cells upon treatment with 200 nM THZ531 for 6 h. Statistical analysis was performed by hypergeometric test using the phyper function of R Stats Package in R Studio Software. **E** GO analysis performed on genes of the overlap shown in B. GO analysis was performed as above. **F** Boxplot comparing RNA expression of MYC between normal adult cerebellum (*n* = 5), normal fetal cerebellum (*n* = 4) and Group 3 MB samples (*n* = 16) derived from the RNA-seq expression dataset EGAD00001004958. Data were analyzed by the Welch’s t-test (**p < 0.01, ***p < 0.001, ****p < 0.0001). **G** Venn diagram showing a significant overlap between genes down-regulated in siMYC-depleted D341 cells and upon treatment with THZ531, as above. Statistical analysis was performed by hypergeometric test using the phyper function of R Stats Package in R Studio Software. **H** GO analysis performed on genes of the overlap shown in G. GO analysis was performed as above

To identify CDK12/13-regulated pathway/s of functional relevance for Group 3 MB, we searched for genes that are both dysregulated in patients and affected by THZ531 treatment. To this aim, we queried RNA-seq data from the International Cancer Genome Consortium (ICGC) and deposited in the European Genome-Phenome Archive (EGA) (https://ega-archive.org; dataset EGAD00001004958; Application #: DACO 5889; Additional file 4: Supplementary Table 3). The transcriptional profiles of 16 Group 3 MB patients were compared to those of healthy controls comprising 4 fetal and 5 adult cerebella, thus taking into account both the embryonic origin of the tumor and the broad spectrum of age at disease onset (from infancy to adolescence) [31]. We identified 4 main clusters of genes that are differentially regulated between Group 3 MB patients and healthy cerebella. Clusters 3 and 4 comprise genes that are down-regulated in MB patients and are enriched in functional terms related to synaptic transmission and nervous system development (Fig. 3A and Additional file 4: Supplementary Table 3), indicating that tumor cells diverge from the physiological differentiation program of the cerebellum. Cluster 1 is enriched in cell cycle genes, which are down-regulated during the transition from fetal to adult cerebellum and reactivated or maintained in Group 3 MB (Fig. 3A and Additional file 4: Supplementary Table 3). By contrast, cluster 2 identified genes that are specifically up-regulated in tumors with respect to both fetal and adult cerebellum. Interestingly, this cluster is enriched in genes related to RNAPII transcription and to the DDR pathway (Fig. 3A and Additional file 4: Supplementary Table 3), two cellular processes related to CDK12/13 function [3, 5, 13].

We found a significant overlap (*n*=108; *p*=5.23^e-39^) between genes down-regulated in Group 3 MB samples and those up-regulated in THZ531-treated cells (Fig. 3B). This set of genes mostly belong to functional categories related to cell migration and metabolic processes (Fig. 3C and Additional file 6: Supplementary Table 5). Likewise, comparison of the genes that are up-regulated in MB patients with those repressed by CDK12/13 inhibition in MB cells yielded a significant overlap (*p* = 1.42^e−11^; Fig. 3D). Annotation of the 175 common genes highlighted functional terms related to key tumorigenic pathways, such as the DDR pathway (i.e. response to radiation, DNA repair, cellular response to DNA damage stimulus), cell cycle (i.e. mitotic cell cycle phase transition, regulation of mitotic cell cycle, cell cycle phase transition) and apoptosis (i.e. apoptotic signaling pathway, negative regulation of programmed cell death; Fig. 3E and Additional file 6: Supplementary Table 5). Importantly, when the comparison was performed with the genes specifically up-regulated in patients classified as Group 3 subtype γ, which express the highest levels of MYC among the Group 3 tumors [31], the overlap was even more significant (*p* = 1.3^e−120^) and was also enriched for genes related to cell cycle and DDR pathway (Supplementary Fig. 3A,B).

Amplification and/or over-expression of the *MYC* oncogene in Group 3 MB patients correlates with increased malignancy and poor prognosis [31]. As expected, we found that MYC expression levels were increased in Group 3 MB patients with respect to both fetal and adult cerebella (Fig. 3F). To identify CDK12/13 target genes whose expression was dependent on MYC, we performed RNA-seq analysis of D341 cells transiently silenced for its expression (Additional file 5: Supplementary Table 4). MYC depletion significantly reduced the proliferation of MYC-high D341 and MED-411 cells (Additional file 2: Supplementary Fig. 4A,B), but it had no effect on MYC-low DAOY cells (Additional file 2: Supplementary Fig. 4C), indicating the dependency of Group 3 MB cells on the expression of this oncogenic transcription factor. RNA-seq analysis indicated that depletion of MYC affected ~ 32% of the genes expressed in D341 cells (Additional file 2: Supplementary Fig. 4D-F). Independent qPCR analysis of 6 arbitrarily selected genes yielded a validation rate of 100% (Additional file 2: Supplementary Fig. 4G), thus confirming the reliability of our bioinformatics analysis. Notably, we observed a significant overlap between MYC- and THZ531-regulated genes (*p* = 1.2^e−14^), with ~ 30% of MYC target genes being also regulated by THZ531 treatment (Additional file 2: Supplementary Fig. 4H). GO analysis of the genes that were down-regulated by both CDK12/13 inhibition and MYC depletion (*n* = 274, *p* = 1.1^e−4^; Fig. 3G) highlighted functional categories related to DNA replication (i.e. regulation of DNA replication, mitotic sister chromatid segregation), cell cycle (i.e. negative regulation of mitotic cell cycle phase transition, meiotic cell cycle) and DDR pathway (i.e. double-strand break repair, via homologous recombination, recombination repair) (Fig. 3H and Additional file 6: Supplementary Table 5). Taken together, these results suggest that the DDR pathway represents a MYC-dependent cellular response that is up-regulated in Group 3 MB patients and is regulated by CDK12/13 activity.

### DDR genes are up-regulated in MYC-high MB patients and cell lines

**Fig. 4:**
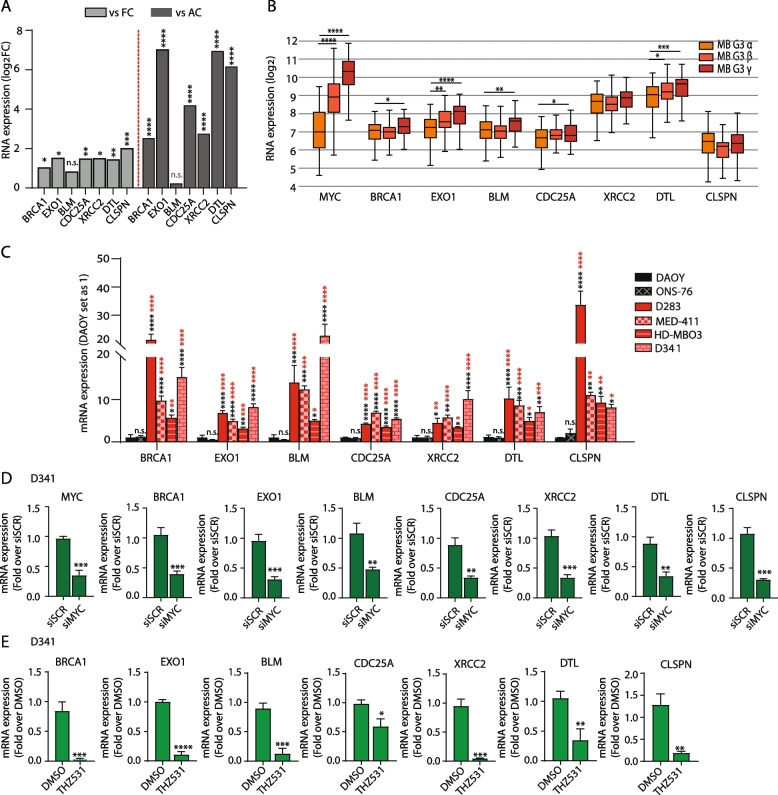
Chemical inhibition of CDK12/13 deeply impaired the DDR in MYC-driven Group 3 MB cells. **A** Bar graphs showing RNA expression levels of the indicated DDR genes in Group 3 MB patients compared to fetal (FC) and adult cerebellum (AC) from EGA RNA-seq expression dataset (EGAD00001004958). Welch's t-test (*p < 0.05, **p < 0.01, ***p < 0.001, ****p < 0.0001); n.s. stands for not significant. **B** Boxplot showing RNA expression of the indicated genes in Group 3 MB patients grouped according to the 3 molecular subtypes (α, β, γ) derived from the publicly available dataset Cavalli. Welch's t-test (*p < 0.05, **p < 0.01, ***p < 0.001, ****p < 0.0001). **C** Bar graphs showing the results of qPCR analyses for the expression of DDR genes in MB cells, as indicated. Data are expressed as fold change relative to DAOY set as 1. mRNA expression is normalized on GAPDH expression. Graphs show the mean ± SD of 3 independent experiments. One-Way ANOVA (*p < 0.05, **p < 0.01, ***p < 0.001, ****p < 0.0001); n.s. stands for not significant. Black and red asterisks indicate statistical analysis performed versus DAOY or ONS-76, respectively. **D** Bar graphs showing the results of qPCR analyses for the expression of MYC and DDR genes in D341 cells depleted for MYC expression by siRNAs-mediated silencing. mRNA expression is normalized on GAPDH expression. Graphs show the mean ± SD of 3 independent experiments. Unpaired t-test (**p < 0.01, ***p < 0.001). **E** Bar graphs showing the results of qPCR analyses for the expression of DDR genes in D341 cells after treatment with 100 nM THZ531 for 8 h. mRNA expression is normalized on GAPDH expression. Graphs show the mean ± SD of 3 independent experiments. Unpaired t-test (***p < 0.001; ****p < 0.0001)

Next, we asked whether up-regulation of DDR genes is a specific feature of Group 3 MB, like its sensitivity to THZ531. Analysis of seven genes belonging to the DDR-related GO terms (*BRCA1*, *EXO1*, *BLM*, *CDC25A*, *XRCC2*, *DTL*, *CLSPN*) revealed that, with the exception of BLM, they are significantly up-regulated in Group 3 MB patients with respect to both fetal and adult cerebella (Fig. 4A). Furthermore, by analyzing the Cavalli dataset [31], we observed that the expression of most DDR genes was higher in Group 3 subtype γ patients, expressing the highest levels of MYC within Group 3 MB, with respect to the α, β subtypes (Fig. 4B). We also analyzed the Pfister’s dataset [40, 41], in which the non-WNT/non-SHH MB patients (*n* = 167) were classified in eight different subtypes (I to VIII) according to the WHO 2021 Classification of Brain Tumours [42]. Expression of most DDR genes was also significantly higher in subtypes II-III-V tumors, which express high MYC levels, with respect to the MYC-low subtypes I-IV-VII tumors (Additional file 2: Supplementary Fig. 5A). Up-regulation (from 2- to > 30-fold) of all the tested DDR genes was also observed in Group 3 MB cells with respect to SHH MB cells (Fig. 4C). Other well-known DDR genes (i.e. *BRCA2*, *RAD51, BARD1* and *WRN*) displayed the same pattern of higher expression in both D283 and D341 with respect to DAOY cells (Additional file 2: Supplementary Fig. 5B). These data suggest that MYC positively controls the expression of multiple DDR genes in Group 3 MB cells. To test this hypothesis, we knocked down its expression in D341, D283 and HD-MBO3 cells. Silencing of MYC in these Group 3 MB cell lines significantly repressed the expression of most of the tested DDR genes (Fig. 4D and Additional file 2: Supplementary Fig. 5C,D), thus confirming their dependency on MYC expression. Moreover, a similar inhibition of DDR gene expression was also achieved by treating these cells with THZ531 (Fig. 4E and Additional file 2: Supplementary Fig. 5E,F), indicating that they are dependent on both MYC expression and CDK12/13 activity.

### CDK12/13 inhibition induces an irreparable DNA damage and massive apoptotic death in MYC-high Group 3 MB cells

**Fig. 5:**
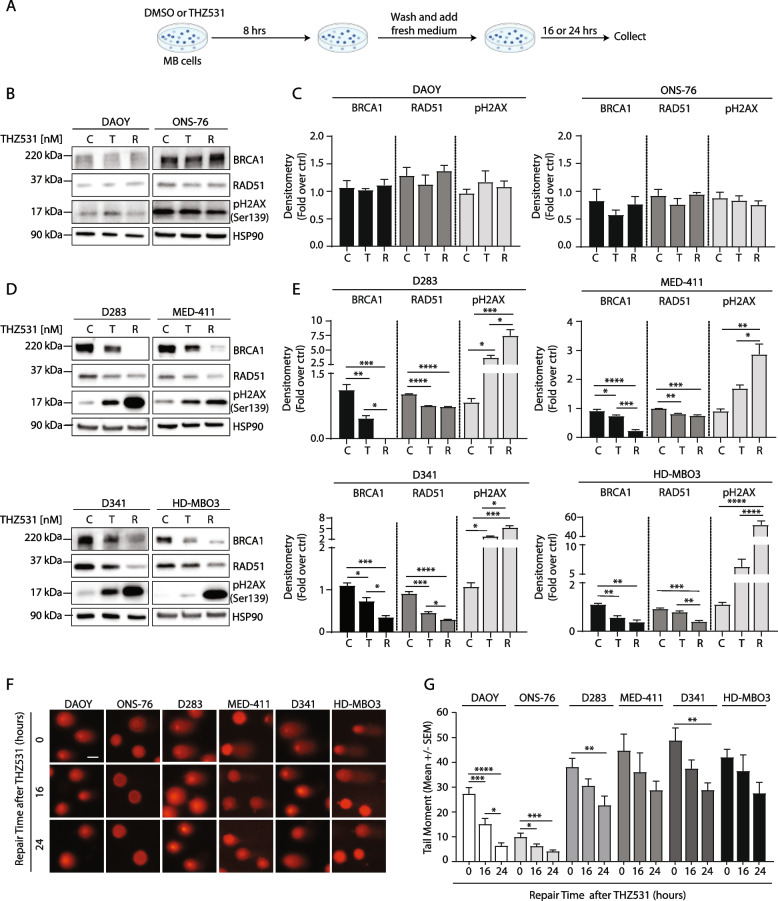
Chemical inhibition of CDK12/13 induces an irreparable DNA damage of MYC-driven Group 3 MB cells. **A** Workflow scheme of the experiment whose results are shown in **B**-**E**. **B-E** WB (**B**,**D**) and densitometric (**C**,**E**,) analysis of DAOY and ONS-76 (**B**,**C**), D283, MED-411, D341 and HD-MBO3 (**D**,**E**) cell lines treated with 100 nM THZ531 for 8 h (T) or DMSO (C), washed and cultured for another 24 h after removal of the treatment (R). HSP90 was used as loading control. Blots in **B** and **D** are representative of 3 independent experiments. Graphs in **C** and **E** show the mean ± SD of 3 independent experiments. Statistical analysis was performed by One-Way ANOVA test (*p < 0.05, **p < 0.01, ***p < 0.001, ****p < 0.0001). **F**-**G** Comet assay (**F**) and relative quantitative analysis (**G**) performed in MB cells treated or not with 100 nM THZ351 for 8 h after which the drug was carefully removed and cells were left to recover for the indicated time. Representative images in **F** are shown from 3 independent experiments. Scale bar: 10 μm. Graph in (**G**) shows data presented as tail moment ± SEM of 3 independent experiments. Statistical analysis was performed by One-Way ANOVA test (*p < 0.05, **p < 0.01, ***p < 0.001, ****p < 0.0001)

The higher expression levels of DDR genes could be related to increased accumulation of endogenous DNA breaks in Group 3 MB cells. To test this hypothesis, we compared the accumulation of DNA breaks by comet assays in Group 3 and SHH MB cells under unperturbed conditions. In line with the hypothesis, Group 3 MB cells displayed significantly higher DNA breaks than SHH cells (Additional file 2: Supplementary Fig. 6A,B). To investigate more in detail the impact of CDK12/13 inhibition on DNA damage repair, we pulsed MB cells with THZ531 (100 nM) for 8 h and let them recover for 24 h (Fig. 5A). In SHH cells, the THZ531 pulse caused a mild (DAOY) or no increase (ONS-76) in phosphorylation of H2AX, a well-established marker of DNA damage [43], which was completely reversed after drug removal (Fig. 5B,C). By contrast, H2AX phosphorylation was more evident in Group 3 MB cells pulsed with THZ531, and the effect of the drug was further augmented during the following recovery step (Fig. 5D,E). Lack of DNA repair during the recovery phase was related to the persistent downregulation of the DDR proteins BRCA1 and RAD51 in THZ531-pulsed Group 3 MB cells (Fig. 5D,E), while these factors were not significantly regulated in SHH cells (Fig. 5B,C). Furthermore, comet assays showed that Group 3 MB cells maintained ~ 60% of the DNA breaks after 24 h of release from THZ531 treatment, wheras SHH cells were able to almost completely recover from the damage (~ 20% residual damage) (Fig. 5F,G).

**Fig. 6:**
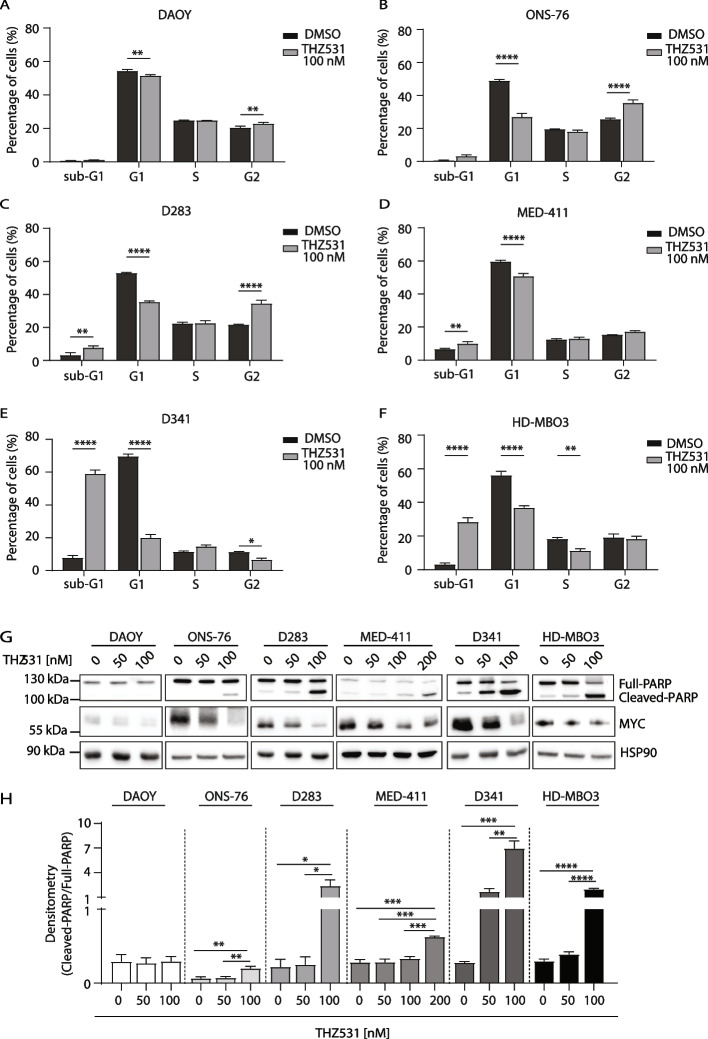
Chemical inhibition of CDK12/13 induces a massive apoptotic death of MYC-driven Group 3 MB cells. **A**-**F** FACS analysis of PI-stained DAOY (**A**), ONS-76 (**B**), D283 (**C**), MED-411 (**D**), D341 (**E**) and HD-MBO3 (**F**) cells following treatment with 100 nM THZ531 for 24 h. Data are represented as mean ± SD of 3 independent experiments. Statistical analysis was performed by One-Way ANOVA test (*p < 0.05, **p < 0.01, ****p < 0.0001). **G** WB analysis of cleaved PARP and MYC expression in MB cell lines treated for 24 h with THZ531, as indicated. Blots are representative of 3 independent experiments. HSP90 was used as loading control. **H** Densitometric analysis of the ratio between cleaved and full-length PARP isoforms relative to WB analysis shown in **G**. Data are represented as mean ± SD of 3 independent experiments. Statistical analysis was performed by One-Way ANOVA test (*p < 0.05, **p < 0.01, ***p < 0.001, ****p < 0.0001)

To test whether persistent DNA damage in the presence of THZ531 affects viability of MYC-high MB cells, we carried out FACS analysis of propidium iodide (PI)-stained cells. Treatment with THZ531 increased the percentage of Group 3 MB cells in the sub-G1 apoptotic fraction (Fig. 6C-F), whereas it had mild (ONS-76) or no effect (DAOY) on SHH cells (Fig. 6A,B). Increased cell death induced by THZ531 in Group 3 MB cells was accompanied by increased cleavage of Poly-ADP-Ribose Polymerase 1 (PARP1; Fig. 6G,H). As shown for viability assays (Fig. 1C and Additional file 2: Supplementary Fig. 1H), the apoptotic effect triggered by THZ531 was directly correlated with MYC expression levels, with D341 and HD-MBO3 showing a higher percentage of cells in sub-G1 fraction (Fig. 6E,F) and increased PARP1 cleavage than D283 and MED-411 cells (Fig. 6G,H). CDK12 was previously reported to regulate MYC expression in other tumour context [44]. Accordingly, we found that that long-term (24 h) CDK12/13 inhibition reduced the expression of MYC in MB cells, with the exception of DAOY cells which is the MB cell line characterized by the lowest expression of this oncogene (Fig. 6G).

Collectively, these results suggest that CDK12/13 activity is required to maintain higher expression of DDR genes and to promote DNA repair proficiency of MYC-high MB cells, while their inhibition triggers DNA lesions and massive apoptotic death.

### CDK12/13 inhibition reduces the RNAPII processivity within DDR genes in MYC-driven Group 3 MB cells

**Fig. 7:**
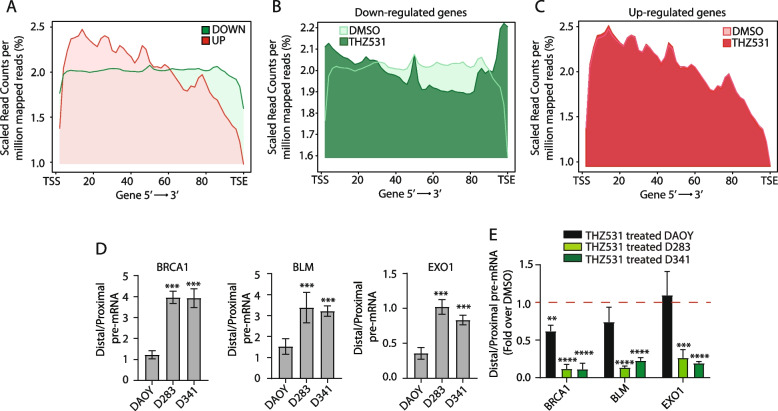
CDK12/13 inhibition reduces the RNAPII processivity within DDR genes in MYC-driven Group 3 MB cells. **A** Metagene plot showing percentages of read counts per million mapped reads distribution within the gene body from transcription start site (TSS) to transcription end site (TES) of down-regulated (DOWN, green line) and up-regulated (UP, red line) genes in untreated D341 cells. **B** Metagene plot showing percentages of read counts per million mapped reads distribution of down-regulated genes in DMSO- (light green line) or THZ531-treated (dark green line) D341 cells. **C** Metagene plot showing percentages of read counts per million mapped reads distribution of up-regulated genes in DMSO- (light red line) and THZ531-treated (dark red line) D341 cells. **D** Bar graphs showing results of qPCR analyses of the ratio between distal and proximal pre-mRNA region of the indicated DDR genes in DAOY, D283 and D341 cells. Data represent the mean ± SD of 3 independent experiments. Statistical analysis was performed by One-Way ANOVA test (***p < 0.001). **E** Bar graphs showing results of qPCR analyses of the ratio between distal and proximal pre-mRNA region of DDR genes in DAOY, D283 and D341 cells after treatment with 100 nM THZ531 for 8 h. Data are expressed as fold change over DMSO-treated cell lines set as 1 (red line). Graphs represent the mean ± SD of 3 independent experiments. Statistical analysis was performed by Unpaired t-test (**p < 0.01, ***p < 0.001, ****p < 0.0001)

Analysis of gene length and expression levels did not reveal significant differences in genes up- or down-regulated upon THZ531 treatment of D341 cells (Additional file 2: Supplementary Fig. 7A,B). However, metagene analysis of reads coverage from the transcription start site (TSS) to the transcription end site (TES) revealed that up- and down-regulated genes display different profiles. Reads coverage was progressively decreased toward the TES of up-regulated genes. By contrast, down-regulated genes showed a homogenous distribution of the reads throughout the transcription unit (Fig. 7A). This analysis suggested an increased processivity of RNAPII (i.e. ability to reach the terminal portion of the gene) within down-regulated genes. Strikingly, the increased reads coverage in the 3’-end of the down-regulated genes was dependent on CDK12/13 activity, as short-term THZ531 treatment strongly reduced it (Fig. 7B), whereas up-regulated genes showed the same profile regardless of CDK12/13 inhibition (Fig. 7C).

Next, we tested whether reduced expression of DDR genes was related to impairment of RNAPII processivity by THZ531. To this aim, we selected MB cell lines with lower (DAOY) and higher (D283 and D341) expression level of DDR genes (Fig. 4B) and we measured RNAPII processivity as the ratio between distal and proximal intronic regions of their pre-mRNAs, with increased processivity yielding a higher distal/proximal ratio [45]. By applying this assay, we observed a higher RNAPII processivity within the *BRCA1*, *EXO1* and *BLM* genes in D283 and D341 MB cells compared to DAOY cells (Fig. 7D). Importantly, the higher processivity entirely relied on CDK12/13 activity, as it was abolished by treatment of D283 and D341 cells with THZ531 for 8 h, whereas the drug elicited mild or no effects in DAOY cells (Fig. 7E). These results suggest that CDK12/13 activity is required to maintain high processivity in a subset of MYC target genes, including DDR genes.

### Combined treatments with THZ531 and DNA damage-inducing agents elicit synergistic cytotoxic effects in MYC-high Group 3 MB cells

**Fig. 8:**
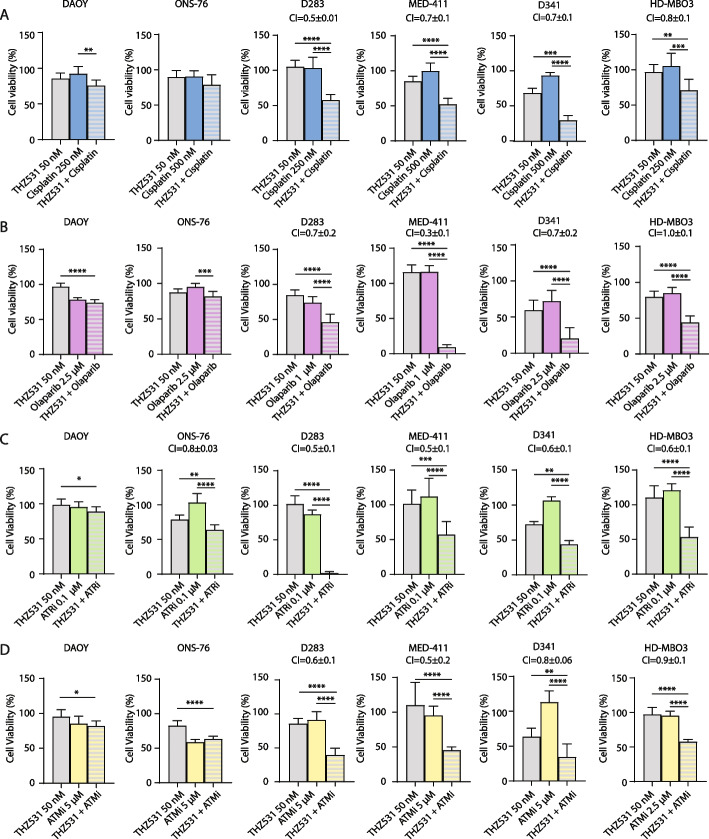
Combined treatments with THZ531 and DNA damage-inducing agents show synergistic effect in suppressing cell viability. **A**-**D** MTS assay of MB cell lines pre-treated for 8 h with a suboptimal dose of THZ531 (50 nM) or DMSO as control, before administration of DNA damage-inducing drugs, such as Cisplatin (**A**), Olaparib (**B**), ATR kinase inhibitor/Ceralasertib (**C**) and ATM kinase inhibitor (**D**). Data represent mean ± SD of 3 independent experiments. Statistical analysis was performed by One-Way ANOVA test (*p < 0.05, **p < 0.01, ***p < 0.001, ****p < 0.0001). The calculated combination index (CI) values for drug combination relative to the individual drugs are presented above the graphs. CI was calculated using CompuSyn software. CI = 1 and < 1 indicates additive effect and synergism, respectively. All results are expressed as the mean ± SD derived from biological triplicates

**Fig. 9:**
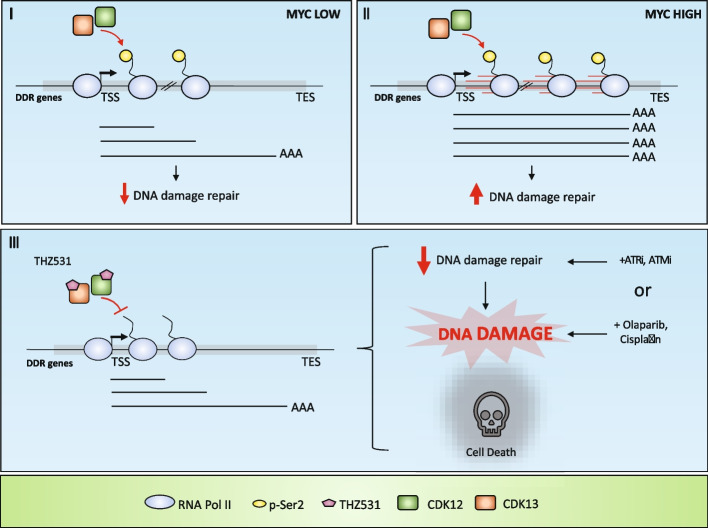
Final model. Schematic model showing the impact of THZ531-mediated inhibition of CDK12/13 on the RNAPII processivity and the expression of DDR genes in MYC-dependent manner; *Abbreviation*: TSS (transcription start site), TES (transcription end site), AAA (poly(A) tail)

Given the effect of THZ531 in reducing the expression of DDR genes in Group 3 MB cells, we hypothesized that the drug could synergize with DNA damage-inducing chemotherapeutic agents. To test this hypothesis, MB cells were pre-treated for 8 h with a suboptimal dose of THZ531 (50 nM; Fig. 1C and Additional file 2: Supplementary Fig. 1H) or DMSO as control, before administration of DNA damage-inducing drugs, such as Cisplatin, Olaparib, ATR and ATM kinase inhibitors. At these doses, THZ531 and the DNA-damaging drugs caused a slight reduction in viability when used as single agents, with the exception of THZ531 on D341 for which we observed ~ 40–50% reduction of viability. Pre-treatment with THZ531 synergically enhanced (combination index < 1) the cytotoxic effects of DNA-damaging drugs in most Group 3 MB cells, whereas SHH MB cells were much less affected by the combined treatments (Fig. 8). These results suggest that pre-treatment with low doses of THZ531 can sensitize MYC-high MB cells to chemotherapeutic treatments (Fig. 9), including Cisplatin that is currently employed in the clinical treatment of MB patients, regardless of the specific subgroup [1].

## Discussion

The *MYC* oncogene is frequently amplified or overexpressed in high-risk Group 3 MB, with high expression of MYC being a prognostic factor of worse outcome [31]. Unfortunately, while the potential of MYC as therapeutic target is well recognized, its pharmacologic inhibition has remained challenging. One possible approach is to target MYC-driven cellular dependencies, such as the transcriptional and/or splicing addiction acquired by MYC-high cells [21]. Herein, we have tested this possibility by evaluating the impact of several transcription and RNA processing inhibitors on the viability of MB cells belonging to subgroups characterized by low (SHH MB) or high (Group 3 MB) expression of MYC. Our screen identified two compounds, Dinaciclib and THZ531, that display increased efficacy in MYC-high Group 3 MB cells. Dinaciclib is a pan-CDK inhibitor, displaying selectivity for CDK1/2/5/9 [35]. In addition, Dinaciclib was also shown to inhibit CDK12 in triple-negative breast cancer cells and to sensitize them to PARP inhibitors in CDK12-dependent manner [38]. On the other hand, THZ531 is a selective inhibitor of the highly homologous CDK12 and CDK13 [13]. Thus, the MYC-dependent susceptibility of Group 3 MB cells to THZ531 and Dinaciclib is likely to rely on inhibition of CDK12 and/or CDK13.

The cytotoxic effect of CDK12/13 inhibition was selective for MYC-high Group 3 MB cells with no or minimal effect in SHH cells. Moreover, sensitivity of MB cell lines to THZ531 was correlated with MYC expression levels. Thus, the up-regulation of MYC in Group 3 MB establishes a dependency on CDK12/13 activity. To elucidate the CDK12/13-dependent transcriptional program of Group 3 MB, we compared the gene signatures that characterize Group 3 MB with that regulated by THZ531. Bioinformatics analyses identified a cluster of genes enriched in functional terms related to transcription and the DDR pathway that is selectively up-regulated in Group 3 MB with respect to both fetal and adult cerebellum and to MB samples expressing lower MYC levels. The DDR pathway was also enriched among the genes repressed by treatment of Group 3 MB cells with THZ531, as well as by depletion of MYC. Moreover, we observed that Group 3 MB cells express higher levels of DDR genes with respect to the THZ531-insensitive SHH MB cells and that CDK12/13 inhibition repressed their expression and the DNA repair in Group 3 MB cells. Thus, up-regulation of the DDR pathway is a key feature of MYC-high MB cells that confer dependency on CDK12/13.

We found that MYC-high Group 3 MB cells concomitantly display high endogenous DNA damage and high expression of DDR genes. This result suggests that MYC-high MB cells are prone to generate DNA lesions and need to reinforce their repair to prevent cell death. Indeed, several observations indicate that MYC regulates transcription of specific gene subsets that concomitantly favour tumour progression and evasion of cell death pathways [46]. The transcriptional amplification generated by MYC imposes a stress to the cell, by promoting collisions between the transcription and replication machineries, increased stalling of the replication fork and DNA lesions [46]. In order to avoid cell death as a consequence of this stress, MYC must set in motion compensatory mechanisms of DNA repair that guarantee cell fitness [46, 47]. The increased expression of DDR genes, such as BRCA1 and RAD51, observed in Group 3 MB cells may represent one of these compensatory mechanisms. However, if their increased expression represents an advantage for MYC-high MB cells, it also generates an actionable vulnerability. Indeed, we found that high expression of DDR genes depends on CDK12/13 and that this generates susceptibility of MYC-high MB cells to inhibition of their activity. Accordingly, low concentrations of THZ531 potently reduced expression of DDR genes and DDR proteins, enhanced DNA damage and induced massive apoptotic cell death in Group 3 MB cells. Thus, our data suggest that the oncogenic function of MYC in Group 3 MB cells can be exploited to selectively kill them through the pharmacologic inhibition of CDK12/13 activity. Since the MYC-N oncogene is also known to positively regulate the expression of DDR genes [48] and Group 4 MB is characterized by MYC-N amplification [31], the increased susceptibility to CDK12/13 inhibitors might also be a feature of Group 4 MB.

We then asked why expression of DDR genes requires CDK12/13 activity in Group 3 MB cells. One feature shared by many DDR genes is their increased length, resulting from numerous long introns [6, 10, 11]. Thus, efficient transcription of DDR genes may require a high elongation rate and processivity, two activities of RNAPII that are regulated by CDK12 and CDK13 through phosphorylation of Serine 2 in its CTD. We found that the CDK12/13-dependent genes display equal representation of reads coverage from the TSS to the TES In MYC-high MB cells. By contrast, up-regulated genes exhibit a progressive decrease in reads coverage towards the end of the transcription unit. These observations suggest that the genes sensitive to CDK12/13 inhibition are transcribed with higher processivity by the RNAPII, which more efficiently reaches the TES. In line with this hypothesis, distal reads coverage in these genes decreases upon treatment with THZ531, resembling the pattern observed in up-regulated genes. Moreover, up-regulated genes, which intrinsically show reduced reads coverage toward the TES, are unaffected by THZ531 treatment. Thus, CDK12/13 activity is crucial to maintain transcription efficiency throughout the gene. Notably, DDR genes showed higher RNAPII processivity in MYC-high MB cells with respect to MYC-low cells and this feature was abolished when CDK12/13 activity was inhibited. Thus, MYC up-regulates the expression of DDR genes, but this regulation requires enhanced RNAPII processivity through CDK12/13 activity in order to reach the end of the gene unit and to yield a full-length transcript. Although previous data suggested that gene length was associated with CDK12-dependency, we observed no significant difference in terms of length and expression between up- and down-regulated genes. Thus, our data indicate that, in MYC-high MB cells, CDK12/13-dependency is established by RNAPII processivity rather than gene length. In line with our results, MYC was recently shown to maintain high RNAPII elongation rate to ensure transcription of distal regions in long genes [49]. To date, MYC-dependent gene expression regulation has been linked to early stages of transcription, which involve CDK7 and CDK9 [50]. Our data now suggest that, once higher RNAPII processivity is established by MYC at the proximal region of genes, proficient transcription becomes dependent on CDK12/13 activity within the gene body.

Mounting evidence indicates that RNA processing regulation is strongly altered in many human tumors, including brain tumors [51, 52]. For instance, inhibitors of PRMT5, an epigenetic regulator that methylates core proteins of the splicing machinery, were shown to exert strong antitumor effects in glioblastoma through pervasive impairment of intron splicing [53]. Likewise, neuroblastoma was reported to overexpress the splicing factor RBM39 and to be highly sensitive to Indisulam [54, 55], a drug that promotes RBM39 degradation [34]. Herein, we demonstrate for the first time that Group 3 MB is highly sensitive to inhibitors of CDK12/13, two transcriptional kinases that are known to couple transcription and RNA processing [3, 6, 7, 10, 11]. Thus, although transcription and splicing represent basic and essential mechanisms for every cell, brain tumors appear to be particularly dependent on these processes and, therefore, highly sensitive to their inhibition by selective drugs. Thus, although the vulnerable step is tumor-specific, transcription and processing of pre-mRNAs represent actionable vulnerabilities for multiple brain cancers.

Despite recent genomic and molecular characterization of the MB subgroups, clinical management of patients is still largely driven by histology, degree of surgical resection and presence or absence of metastases [1]. The present study reveals the selective chemotherapeutic efficacy of THZ531 in MYC-high MB, thus further highlighting the importance to diversify the treatments on the basis of the genomic and molecular profiles of the tumour, as already in use for other cancer types (i.e. breast and colon cancers). Given the functional relevance of the DDR pathway in Group 3 MB and its dependency on CDK12/13 activity, we asked whether treatment with THZ531 could sensitize Group 3 MB cells to DNA damaging agents. Cisplatin is a DNA alkylating drug currently in use for treatment of MB patients [1]. Our data indicate that combined treatment with THZ531 significantly ameliorates the cytotoxic effect of Cisplatin in MYC-high MB cells. Moreover, we found that THZ531 synergically enhanced the cytotoxic activity of targeted DNA damage-inducing agents, such as Olaparib, ATR and ATM inhibitors, that are in use or being evaluated in clinical trials for other cancer types. Thus, our study suggests that pharmacologic inhibition of CDK12/13 activity, for which new and more selective inhibitors are being developed [56–58], represents a promising therapeutic approach for MYC-high Group 3 MB, either as single agent or in combination with DNA damage-inducing drugs.

## Conclusions

Our study demonstrated that: *i)* THZ531, an inhibitor of the CDK12/13, is highly selective for MYC-high MB cells with respect to MYC-low MB cells; *ii*) high expression of DDR genes in MYC-high MB cells depends on MYC- and CDK12/13-dependent RNA polymerase processivity; *iii)* pharmacological inhibition of CDK12/13 impairs the DDR and induces irreparable DNA damage exclusively in MYC-high MB cells. Lastly, treatment with THZ531 synergically enhanced the cytotoxic effects of DNA damage-inducing agents in Group 3 MB cells. Thus, our study suggests that CDK12 and CDK13 are actionable targets in MYC-high MB and may pave the ground for pre-clinical studies aimed at evaluating the inhibition of both kinases as clinical opportunities for high-risk MB patients in the near future.

### Supplementary Information

**Additional file 1:** **Supplementary Table 1.** List of primers, siRNA and shRNA sequences used in this study.

**Additional file 2:** **Supplementary Figures 1–7.** Supplementary figures and their figure legends.

**Additional file 3:** **Supplementary Table 2.** RNA-seq expression data of DMSO- and THZ531-treated D341 MB cells.

**Additional file 4:** **Supplementary Table 3.** RNA-seq expression data of MB Group 3 patients and healthy adult and fetal cerebella (Dataset: EGAD0000100495). Gene ontology analysis and lists of genes included in the enriched GO terms.

**Additional file 5: Supplementary Table 4.** RNA-seq expression data of siMYC and siSCR D341 MB cells.

**Additional file 6:** **Supplementary Table 5.** Gene ontology analysis and lists of genes included in the enriched GO terms.

## Data Availability

All data generated or analyzed during this study are included in this article. RNA-seq data are available in GEO database (accession number GSE225375).

## Abbreviations

MB: Medulloblastoma
CDKs: Cyclin-dependent kinases
WNT: Wingless
SHH: Sonic Hedgehog
TA-CDKs: Transcription-associated cyclin-dependent kinases
CTD: Carboxyl-terminal domain
RNAPII: RNA polymerase II
DDR: DNA damage response
RBP: RNA binding protein
RNA-Seq: RNA sequencing
snRNPs: Small nuclear ribonucleoproteins
qPCR: Real time PCR
ICGC: International Cancer Genome Consortium
EGA: European Genome-Phenome Archive
TSS: Transcription star site
TES: Transcription end site
DEGs: Differentially expressed genes (DEGs)
GO: Gene ontology
FC: Fetal cerebellum
AC: Adult cerebellum
