# Supplementary material for: MYC up-regulation confers vulnerability to dual inhibition of CDK12 and CDK13 in high-risk Group 3 medulloblastoma
Source: J Exp Clin Cancer Res. 2023 Aug 21;42:214. doi: 10.1186/s13046-023-02790-2 (PMC10440921; doi:10.1186/s13046-023-02790-2)
Supplement: Supplementary file 2 — Additional file 2: Supplementary Figures 1–7. Supplementary figures and their figure legends. [file 13046_2023_2790_MOESM2_ESM.pdf]

MYC up-regulation confers vulnerability to dual inhibition of CDK12 and CDK13 in high-risk Group 3 medulloblastoma

Consuelo Pitolli[1], Alberto Marini[1,2], Marika Guerra[1], Marco Pieraccioli[1,2], Veronica Marabitti[3,4], Fernando Palluzzi[5], Luciano Giacò[5], Gianpiero Tamburrini[1,6], Francesco Cecconi[3,7,8], Francesca Nazio[3,4], Claudio Sette[1,2*] and Vittoria Pagliarini[1,2*]

[1] Department of Neuroscience, Section of Human Anatomy, Catholic University of the Sacred Heart, 00168 Rome, Italy

[2] GSTEP-Organoids Research Core Facility, IRCCS Fondazione Policlinico Universitario Agostino Gemelli, 00168 Rome, Italy

[3] Department of Pediatric Hemato-Oncology and Cell and Gene Therapy, Bambino Gesù Children's Hospital, IRCCS, Rome, Italy

[4] Department of Biology, University of Rome Tor Vergata, Rome, Italy

[5] Bioinformatics Research Core Facility, Gemelli Science and Technology Park (GSTeP), IRCCS Fondazione Policlinico Universitario Agostino Gemelli, 00168 Rome, Italy

[6] Pediatric Neurosurgery, IRCCS Fondazione Policlinico Universitario Agostino Gemelli, 00168 Rome, Italy

[7] Department of Basic Biotechnological Sciences, Intensive Care and Perioperative Clinics Research, Catholic University of the Sacred Heart, 00168 Rome, Italy;

[8] Unit of Cell Stress and Survival, Danish Cancer Society Research Center, Copenhagen, Denmark

* To whom correspondence should be addressed. Email: claudio.sette@unicatt.it and vittoria.pagliarini@unicatt.it

Present Address:

[Fernando Palluzzi], Integrated Omics Department, Novo Nordisk, 2860 Søborg, Denmark

Additional File 2. Supplementary Figures 1-7

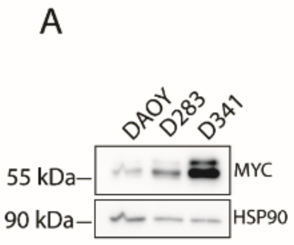

B

		LogIC50 [M]± SE [M]		
Inhibitor	*Targets*	*DAOY*	*D283*	*D341*
EPZ015666	PRMT5	-2.0 ± 1.6	-3.9 ± 0,8	-4.7 ± 0,3
Pladienolide	SF3B1	-8.6 ± 0.1	-9.0 ± 0.04	-8.9 ± 0.05
Indisulam	RBM39	-5.9 ± -0.4	-5.2 ± -0.8	-5.7 ± -1.4
Dinaciclib	pan-CDKs	-7.5 ± 0.6	-8 ± 112.3	-8 ± 66.6
THZ1	CDK7-12-13	-7.3 ± 0.07	-7.3 ± 0.05	-7.5 ± 0.04
THZ531	CDK12-13	-6.4 ± 0.05	-7.0 ± 27.8	-7.3 ± 0.008
SR4835	CDK12-13	-6.8 ± 0.02	-7.1 ± 0.01	-7.1 ± 0.01

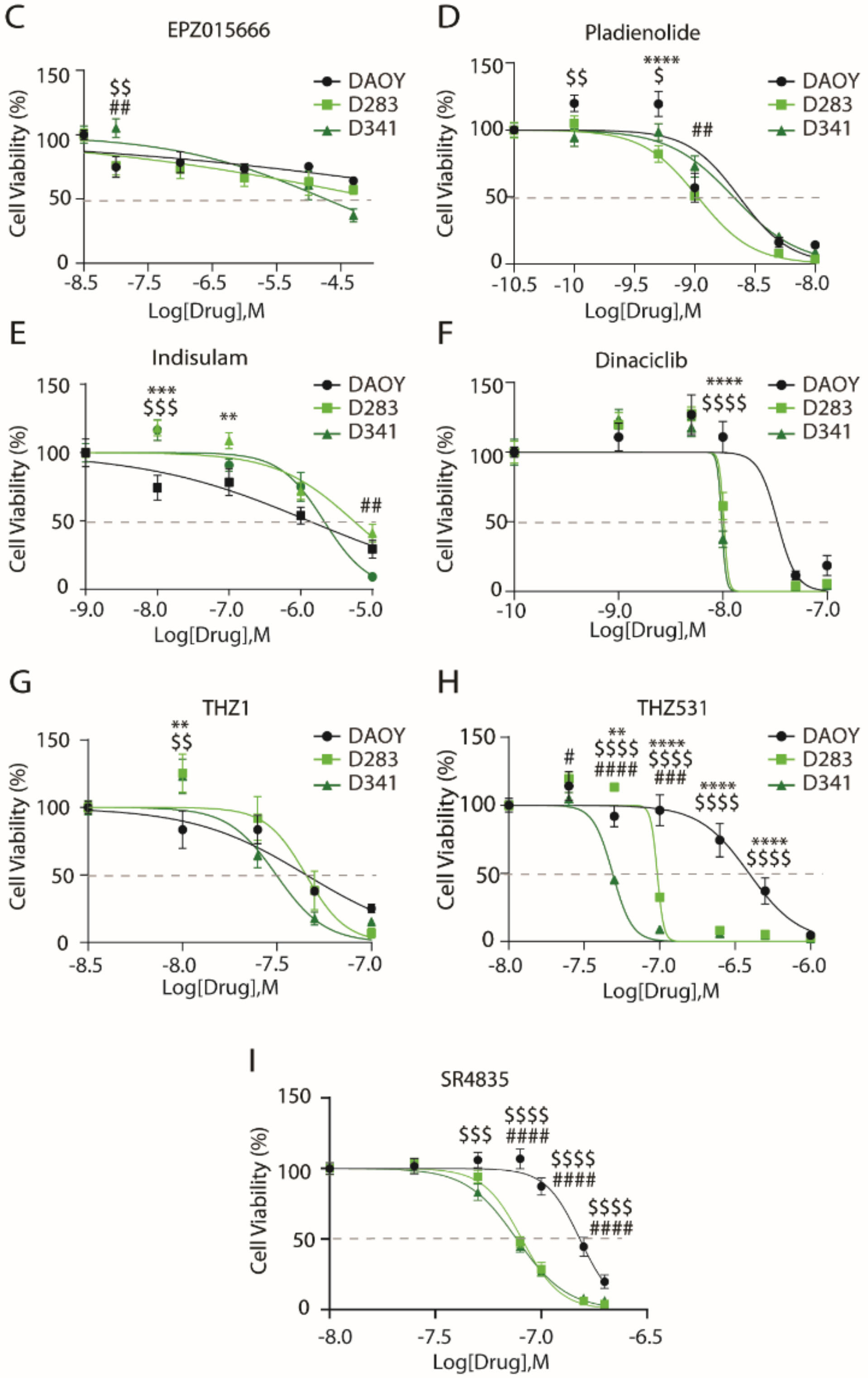

Supplementary Figure 1 ***MYC confers susceptibility of Group 3 MB cells to pharmacological inhibition of CDK12/13.*** **A** WB analysis of MYC expression level in MB cells, as indicated. HSP90 was used as loading control. Blots are representative of 3 independent experiments. **B** IC50 values of splicing and transcription inhibitors tested in MB cells. **C**-**I** MTS assay of MB cells treated for 72 hrs with increasing doses of EPZ015666 (**C**), Pladienolide B (**D**), Indisulam (**E**), Dinaciclib (**F**), THZ1 (**G**), THZ531 (**H**) and SR4835 (**I**). Data represents mean ± SEM of 3 independent experiments. Statistical analysis was performed by Two-Way ANOVA test (**p<0.01, ***p<0.001, ****p<0.0001 (DAOY *vs* D283); $p<0.05, §§p<0.01, §§§p<0.001, §§§§p<0.0001 (DAOY *vs* D341); #p<0.05, ##p<0.01, ###p<0.001, ####p<0.0001 (D283 *vs* D341)).

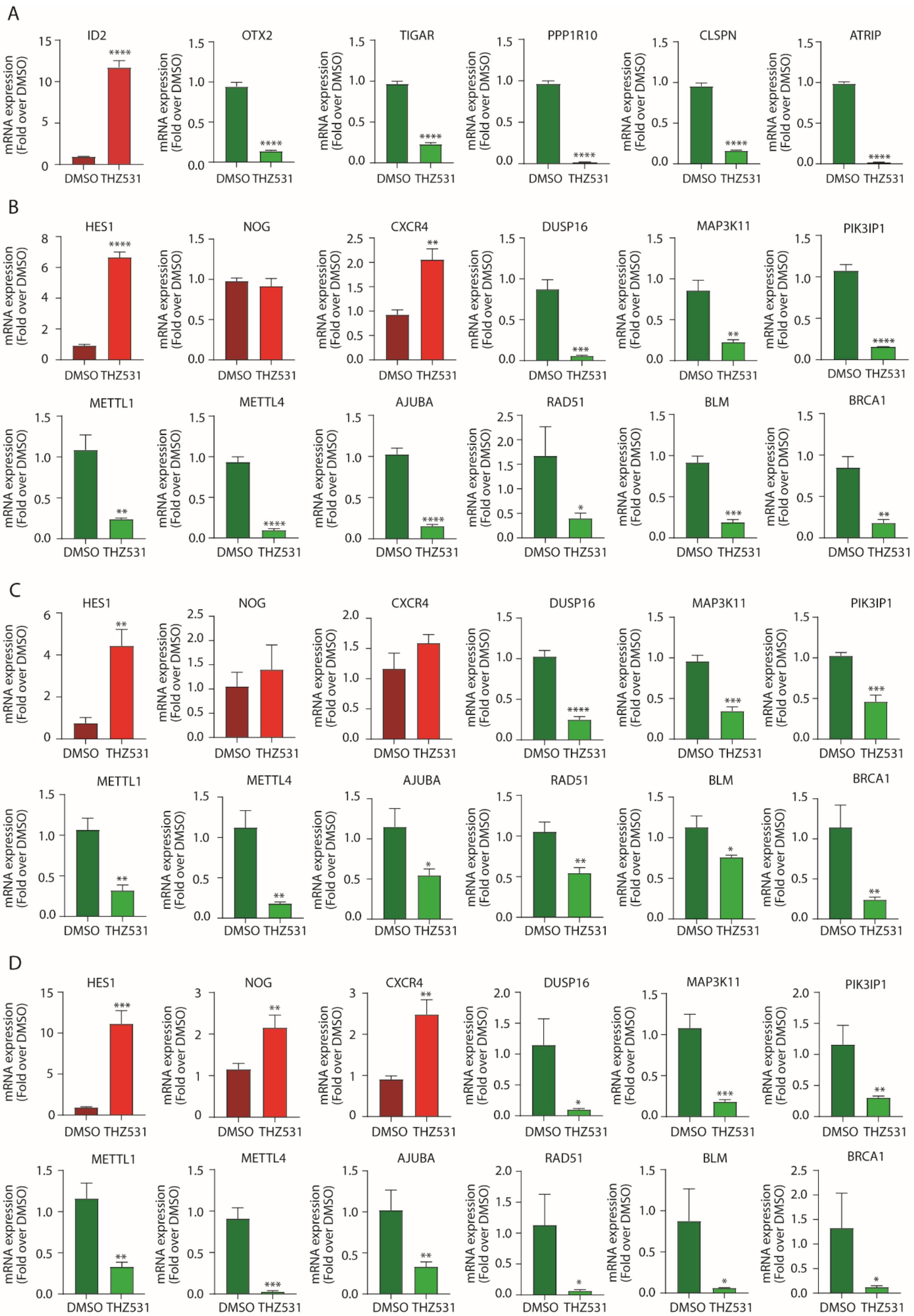
A
ID2
OTX2
TIGAR
PPP1R10
CLSPN
ATRIP
B
HES1
NOG
CXCR4
DUSP16
MAP3K11
PIK3IP1
METTL1
METTL4
AJUBA
RAD51
BLM
BRCA1
C
HES1
NOG
CXCR4
DUSP16
MAP3K11
PIK3IP1
METTL1
METTL4
AJUBA
RAD51
BLM
BRCA1
D
HES1
NOG
CXCR4
DUSP16
MAP3K11
PIK3IP1
METTL1
METTL4
AJUBA
RAD51
BLM
BRCA1
mRNA expression
(Fold over DMSO)
DMSO
THZ531

Supplementary Figure 2 ***Pharmacologic inhibition of CDK12/13 reprograms the transcriptome of MYC-driven Group 3 MB cells.*** Bar graphs showing the results of qPCR analyses for the expression of the indicated genes in D341 (**A**), D283 (**B**), MED-411 (**C**) and HD-MBO3 (**D**) MB cell lines. mRNA expression is normalized on GAPDH expression. Graphs show the mean ± SD of 3 independent experiments. Statistical analysis was performed by unpaired two-tailed Student's t test (*p<0.05, **p<0.01, ***p<0.001, ****p<0.0001).

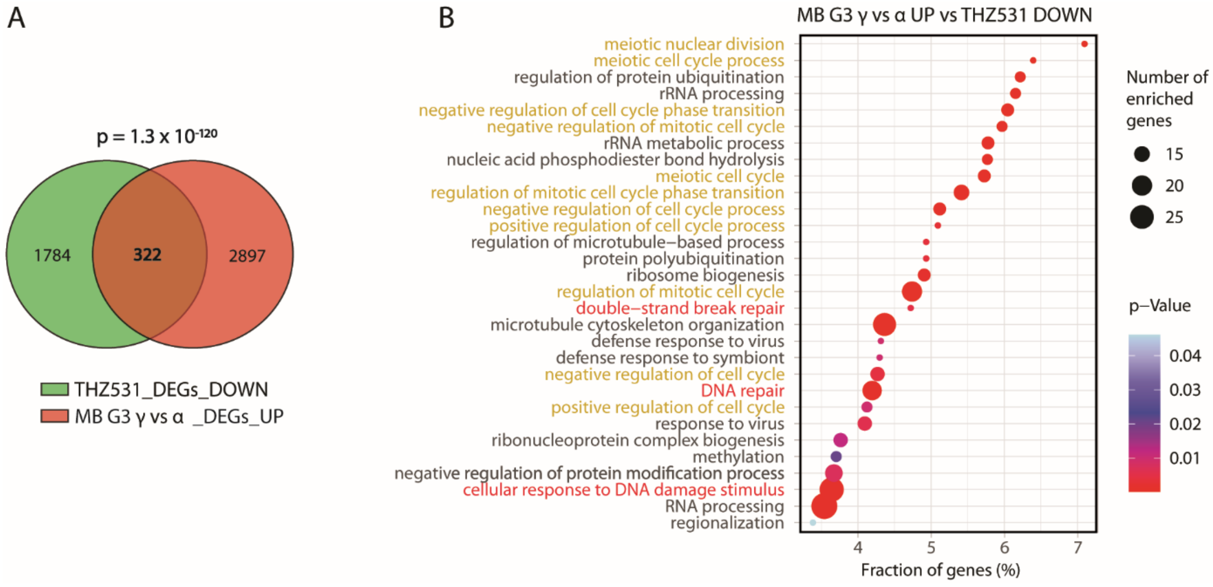

Supplementary Figure 3 ***CDK12/13 inhibition impairs the DNA damage response in high-MYC Group 3 MB patients*****. A** Venn diagram showing a significant overlap between genes up-regulated in high-MYC Group 3 MB patients (analysis has been performed by comparing GE of Group 3 MB patients belonging to subtype γ with those of subtype α, both derived from the publicly available dataset Cavalli) and genes down-regulated in THZ531-treated D341 cells. Statistical analysis was performed by hypergeometric test using the phyper function of R Stats Package in R Studio Software. **B** GO terms enriched for genes of the overlap showed in **A**. GO analysis was performed by using TopGO package in R Studio Software.

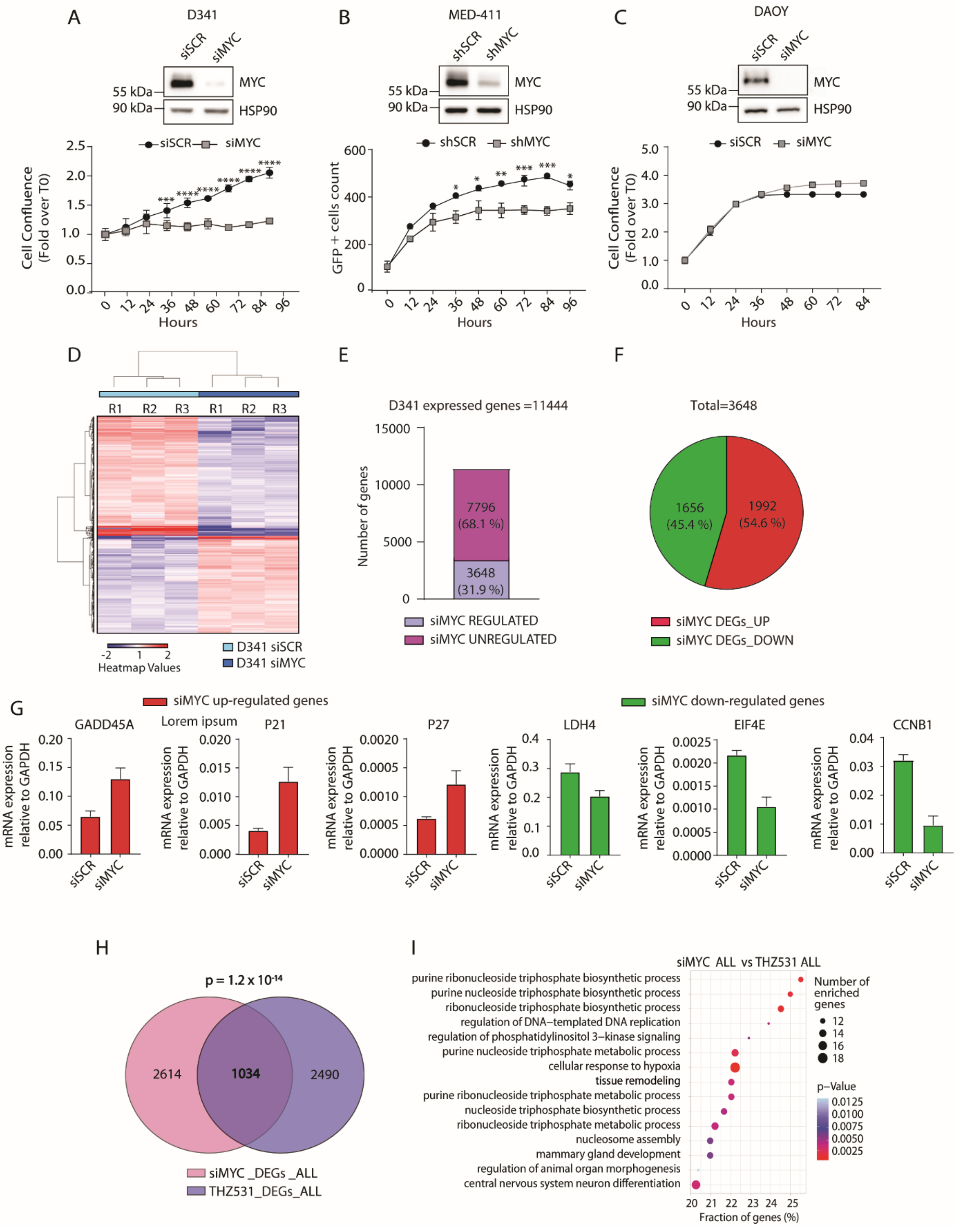

Supplementary Figure 4 ***MYC-driven Group 3 MB cell lines are highly dependent on high MYC expression level for their viability.*** **A** Percentage of cell confluence in D341 cells silenced (siMYC) or not (siSCR) for MYC expression, as indicated. Data are expressed as mean ± SEM of 3 independent experiments. Statistical analysis was performed by Two-Way ANOVA test (***p<0.001, ****p<0.0001). MYC expression in D341 cells silenced (siMYC) or not (siSCR) for MYC expression

was evaluated by WB analysis. HSP90 was used as loading control. **B** Count of GFP-positive MED-411 cells infected with pLVTHM-shSCR or pLVTHM-shMYC lentiviral vectors. Data are expressed as mean ± SEM of 3 independent experiments. Statistical analysis was performed by Two-Way ANOVA test (*$p<0.05$, **$p<0.01$, ***$p<0.001$). ShRNA-mediated downregulation of MYC expression was evaluated by WB analysis. **C** Percentage of cell confluence in DAOY cells silenced (siMYC) or not (siSCR) for MYC expression, as indicated. Data are expressed as mean ± SEM of 2 independent experiments. SiRNA-mediated down-regulation of MYC expression in DAOY cells was evaluated by WB analysis. **D** Heat map of gene expression upon siRNA-mediated depletion of MYC expression (siMYC) in D341 cell line. FC>1.5, p-Adj<0.05. **E** Bar graph shows the percentage of regulated and unregulated genes in D341 cells upon silencing of MYC expression, as above. **F** Pie chart of up-regulated and down-regulated genes (%) in D341 cells upon siRNA-mediated depletion of MYC, as above. **G** Bar graphs showing the results of qPCR analyses for the expression of MYC target genes performed in D341 cells silenced (siMYC) or not (siSCR) for MYC expression, as indicated. Data are normalized on GAPDH mRNA levels and represented as mean ± SD of 2 independent experiments. **H** Venn diagram showing a significant overlap between genes down-regulated in D341 cells upon siRNA-mediated depletion of MYC (siMYC) and genes down-regulated in THZ531-treated D341 cells. Statistical analysis was performed by hypergeometric test using the phyper function of R Stats Package in R Studio Software. **I** GO terms enriched for genes of the overlap showed in H. GO analysis was performed by using TopGO package in R Studio Software.

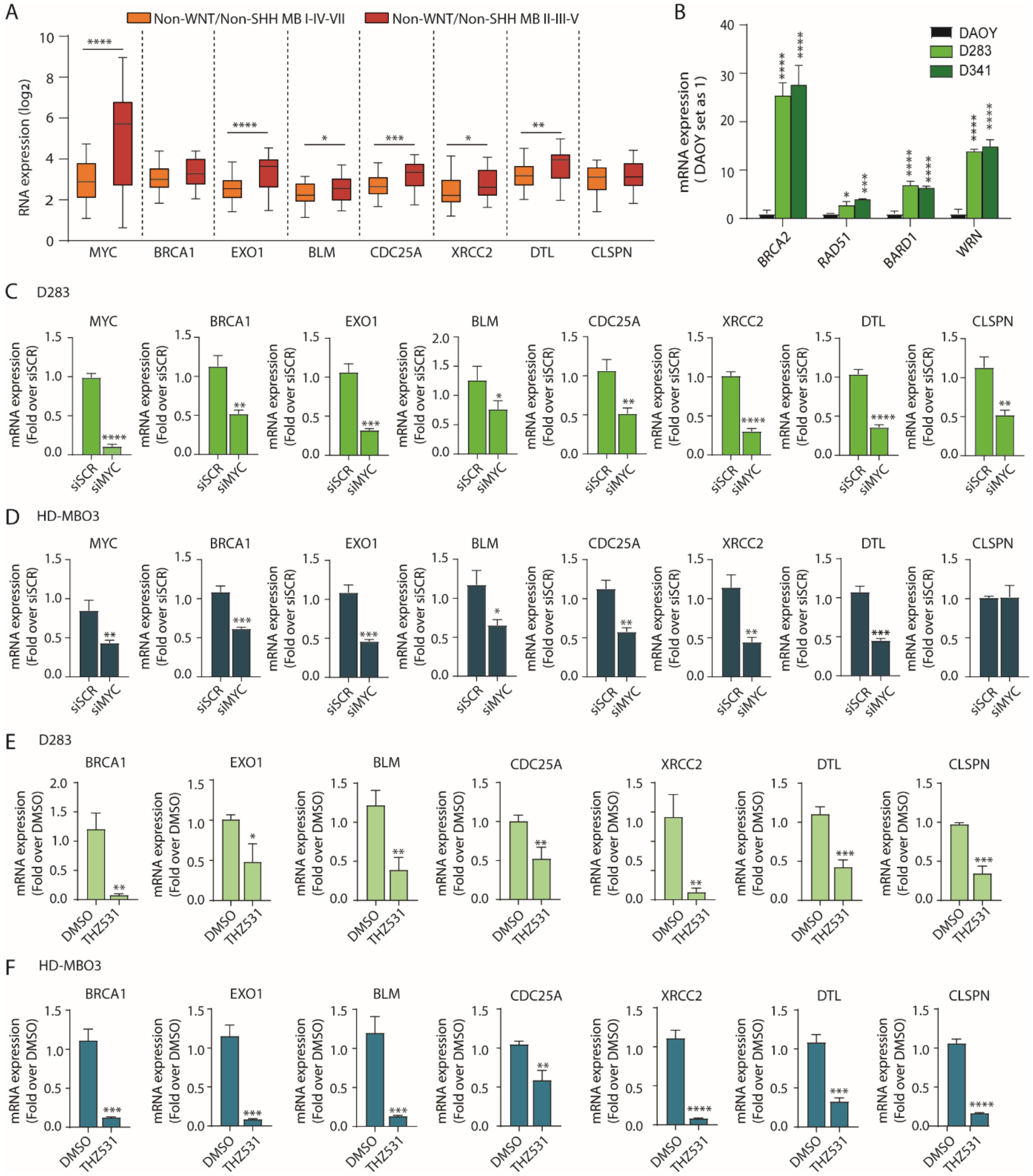

Supplementary Figure 5 ***MYC-driven Group 3 MB cell lines showed higher expression levels of DDR genes compared to SHH cells.*** **A** Boxplot showing RNA expression of the indicated genes in non-WNT/non-SHH MB patients grouped according to the 8 molecular subtypes (I-VIII) derived from the publicly available dataset Pfister. Subtypes I-IV-VII grouped non-WNT/non-SHH MB patients characterized by low level of MYC expression, while subtypes II-III-V grouped non-WNT/non-SHH MB patients characterized by high level of the oncogene (Northcott *et al.*, Nature 2017; Sharma *et al.*, Acta Neuropathol. 2019). Welch's t-test (*p<0.05, **p<0.01, ***p<0.001, ****p<0.0001). **B** Bar graphs showing the results of qPCR analyses for the expression of DDR genes in DAOY, D283 and D341 cells, as indicated. Data are expressed as fold change relative to DAOY set as 1. mRNA expression is

normalized on GAPDH expression. Graphs show the mean ± SD of 3 independent experiments. Statistical analysis was performed by One-Way ANOVA (*p<0.05, ***p<0.001, ****p<0.0001). **C**-**D** Bar graphs showing the results of qPCR analyses for the expression of MYC and DDR genes in D283 (**C**) and HD-MBO3 (**D**) cells depleted for MYC expression by siRNAs-mediated silencing. mRNA expression is normalized on GAPDH expression. Graphs show the mean ± SD of 3 independent experiments. Unpaired t-test (*p<0.05, **p<0.01, ***p<0.001, ****p<0.0001). **E**-**F** Bar graphs showing the results of qPCR analyses for the expression of DDR genes in D283 (**E**) and HD-MBO3 (**F**) cells after treatment with 100 nM THZ531 for 8 hrs. mRNA expression is normalized on GAPDH expression. Graphs show the mean ± SD of 3 independent experiments. Unpaired t-test (*p<0.05, **p<0.01, ***p<0.001, ****p<0.0001).

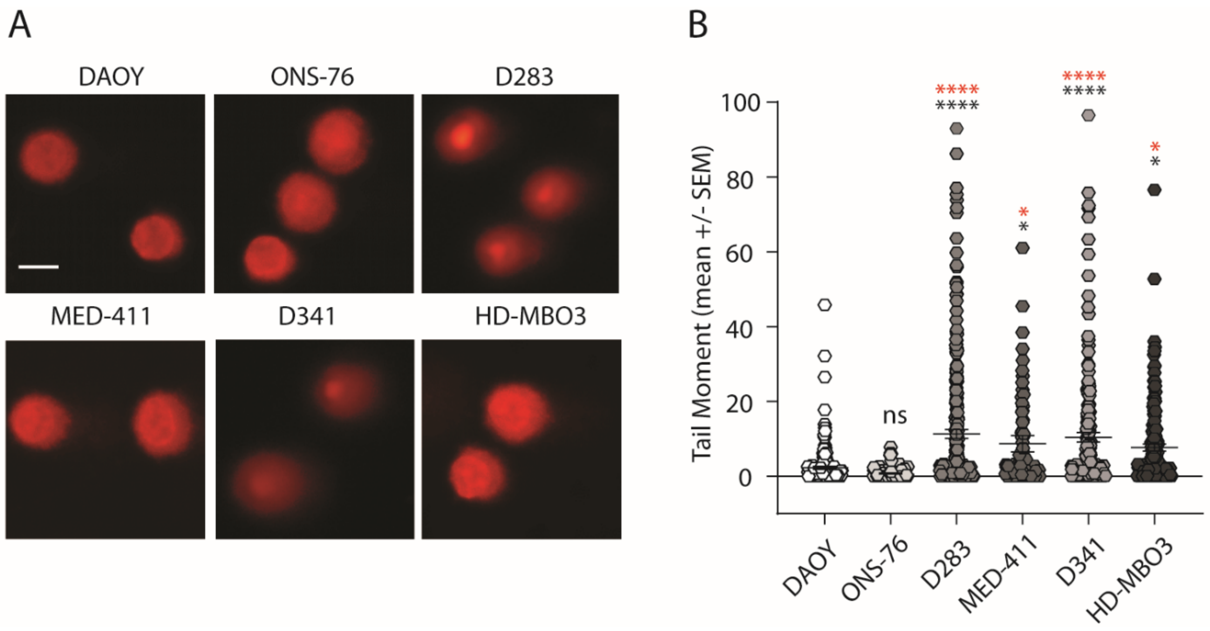

Supplementary Figure 6 ***MYC-driven Group 3 MB cell lines showed higher number of DNA breaks compared to SHH cells.*** **A**-**B** Comet assay (**A**) and relative quantitative analysis (**B**) performed in MB cells, as indicated. Representative images in **A** are shown from 3 independent experiments. Scale bar: 10 μm. Graph in **B** shows data presented as tail moment ± SEM. Statistical analysis was performed by One-way ANOVA (*p<0.05, ****p<0.0001). ns stands for not significant. Black and red asterisks indicate statistical analysis performed versus DAOY or ONS-76, respectively.

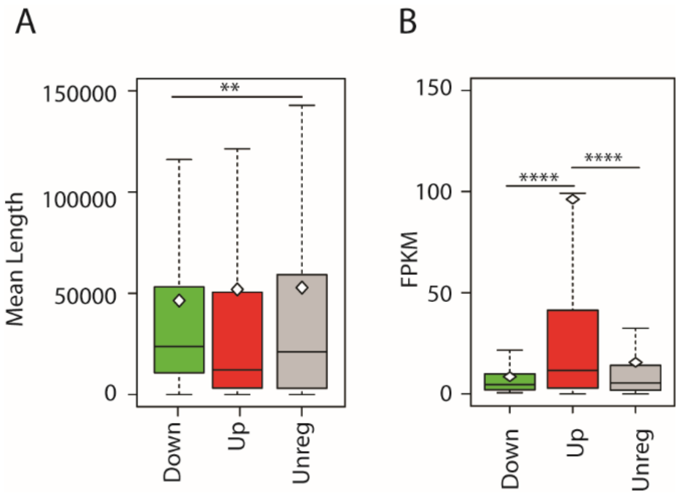

Supplementary Figure 7 ***Analysis of gene length and expression levels did not reveal significant differences in genes up- or down-regulated upon THZ531 treatment of D341 cells*.** **A**-**B** Boxplot graphs representing gene length (**A**) and FPKM (**B**) relative to down-regulated (Down), up-regulated (Up) and unregulated (Unreg) genes in D341cells upon treatment with 200nM THZ531 for 6 hrs. P-values refer to the differences between mean values estimated with a t test (**p<0.01, ****p<0.0001). Median as reported as black line.
